# Exploring the Functional Basis of Epigenetic Aging in Relation to Body Fat Phenotypes in the Norfolk Island Cohort

**DOI:** 10.3390/cimb45100497

**Published:** 2023-09-27

**Authors:** Thao Van Cao, Heidi G. Sutherland, Miles C. Benton, Larisa M. Haupt, Rodney A. Lea, Lyn R. Griffiths

**Affiliations:** 1 Centre for Genomics and Personalised Health, School of Biomedical Sciences, Queensland University of Technology (QUT), Kelvin Grove, QLD 4059, Australia; thaovan.cao@hdr.qut.edu.au (T.V.C.); heidi.sutherland@qut.edu.au (H.G.S.); miles.benton84@gmail.com (M.C.B.); larisa.haupt@qut.edu.au (L.M.H.); lyn.griffiths@qut.edu.au (L.R.G.); 2 ARC Training Centre for Cell and Tissue Engineering Technologies, Queensland University of Technology (QUT), Kelvin Grove, QLD 4059, Australia; 3 Max Planck Queensland Centre for the Materials Sciences of Extracellular Matrices, Queensland University of Technology (QUT), Kelvin Grove, QLD 4059, Australia

**Keywords:** Norfolk Island, epigenetics, aging, DNA methylation, gene expression, obesity

## Abstract

DNA methylation is an epigenetic factor that is modifiable and can change over a lifespan. While many studies have identified methylation sites (CpGs) related to aging, the relationship of these to gene function and age-related disease phenotypes remains unclear. This research explores this question by testing for the conjoint association of age-related CpGs with gene expression and the relation of these to body fat phenotypes. The study included blood-based gene transcripts and intragenic CpG methylation data from Illumina 450 K arrays in 74 healthy adults from the Norfolk Island population. First, a series of regression analyses were performed to detect associations between gene transcript level and intragenic CpGs and their conjoint relationship with age. Second, we explored how these age-related expression CpGs (eCpGs) correlated with obesity-related phenotypes, including body fat percentage, body mass index, and waist-to-hip ratio. We identified 35 age-related eCpGs associated with age. Of these, ten eCpGs were associated with at least one body fat phenotype. Collagen Type XI Alpha 2 Chain (*COL11A2*), Complement C1s (*C1s*), and four and a half LIM domains 2 (*FHL2*) genes were among the most significant genes with multiple eCpGs associated with both age and multiple body fat phenotypes. The *COL11A2* gene contributes to the correct assembly of the extracellular matrix in maintaining the healthy structural arrangement of various components, with the *C1s* gene part of complement systems functioning in inflammation. Moreover, *FHL2* expression was upregulated under hypermethylation in both blood and adipose tissue with aging. These results suggest new targets for future studies and require further validation to confirm the specific function of these genes on body fat regulation.

## 1. Introduction

Biological aging is a complex process that involves changes in many molecular systems, including cellular senescence, genomic instability, telomere attrition, epigenetic alteration, and loss of proteostasis [1]. In humans, these changes lead to an increase in the risk of many complex diseases such as cancer, cardiovascular disease, and Alzheimer’s disease [2,3,4]. As aging is a complex process with many biological systems changing over time, understanding the fundamental molecular mechanisms involved may lead to a better understanding of how to enhance the quality of life into old age.

The rate of biological aging varies substantially among humans, and the variation is due to the complex interplay of genetic profiles, environmental exposures, and epigenetic modifications. DNA methylation is a particular epigenetic modification involving the addition of a methyl group to cytosine-phosphate-guanine (CpG) sites. The addition or loss of methylation is to a large extent developmentally regulated, but can also be influenced by environmental exposures [5]. Moreover, methylation profiles at specific loci have been shown to change over time as a result of epigenetic drift [6]. Changes in DNA methylation levels can modulate gene expression levels due to the disruption of transcription factor binding or the recruitment of repressors [7,8]. While it is widely accepted that there is a negative correlation between methylation at the transcription start site and gene expression [9,10], studies have also shown a positive correlation between methylation in the gene body and an influence on expression [11]. DNA methylation could also control gene expression from a long distance away from promoters through its effect on distal regulatory elements such as enhancers. Enhancers can control gene expression by the formation of chromatin loops [12]. The methylation patterns on these regions were generally unmethylated [13]. Studies showed that the methylation patterns on enhancers could be used to classify different cells such as normal versus tumour cells [14].

Many studies have been performed to identify associations between DNA methylation and chronological aging. The Epigenome-wide Association Studies (EWAS) Atlas summarizes all EWASs conducted on various phenotypes [15]. Among them, the EWAS Atlas contains a list of more than 20,000 methylation CpGs annotated to 10,837 genes associated with aging in DNA collected from blood samples [15]. Age-related CpGs can also be considered a biomarker of aging from which biological age can be estimated (i.e., epigenetic clocks). By calculating epigenetic age acceleration, these clocks have been shown to accurately predict human lifespan and a large range of morbidities [3,4].

The mechanism by which these age-related CpGs affect the expression of the linked genes is not fully understood. To address this issue, Peters et al. conducted a meta-analysis of human peripheral blood in 14,983 individuals of European ancestry and identified 1497 genes differentially expressed with chronological age [16]. Of these, 1248 genes showed a potential enrichment of methylation sites (1 to 154 CpGs per gene) associated with chronological age and gene expression. These genes were enriched in various pathway clusters, including DNA replication, elongation, and mismatch repair; fatty acid metabolism; peroxisome activity; RNA metabolism; ribosome biogenesis; and purine metabolism. The study showed the link between the association of CpG methylations and gene expression (or eCpGs) with the change in many aging pathways.

Changes in body composition in terms of lipid metabolism and storage are essential factors in age-related diseases [17,18]. Excess body fat (especially visceral) is associated with decreased life expectancy [19]. Trim et al. showed similarities in the immunological profiles of aging and excess body fat [20], suggesting overlapping pathways between immunosenescence and obesity. Later, in 2019, a review paper discussed the correlation of aging and obesity in terms of nine critical hallmarks of the aging process [21], which also indicated a strong link between changes in body composition and aging. A recent meta-analysis using methylation and transcription data showed a younger epigenetic age with higher fitness level [22]. Therefore, understanding the pathways linking body fat composition and aging might provide better insight into the mechanisms that regulate the maintenance of good health.

Norfolk Island (NI) has been part of a long-term health study over the past 20 years, with a well-documented population history and known ancestry [23]. The island was founded by ten Bounty Mutineers and six Tahitian wives [24]. Since then, immigration has been limited due to its geographic location and restrictions. Due to the small number of founders and the isolated environment, the island provides less variation in environmental factors and, therefore, reduced genomic diversity [24]. Since aging involves complex changes affected by genetics together with the impact of the environment, the use of an isolated population could help to minimise the variation within the population.

This exploratory study aims to identify interactions between genes affected by age-related DNA methylation and its impact on gene expression and obesity-related phenotypes using the isolated NI population cohort.

## 2. Materials and Methods

**Figure 1:**
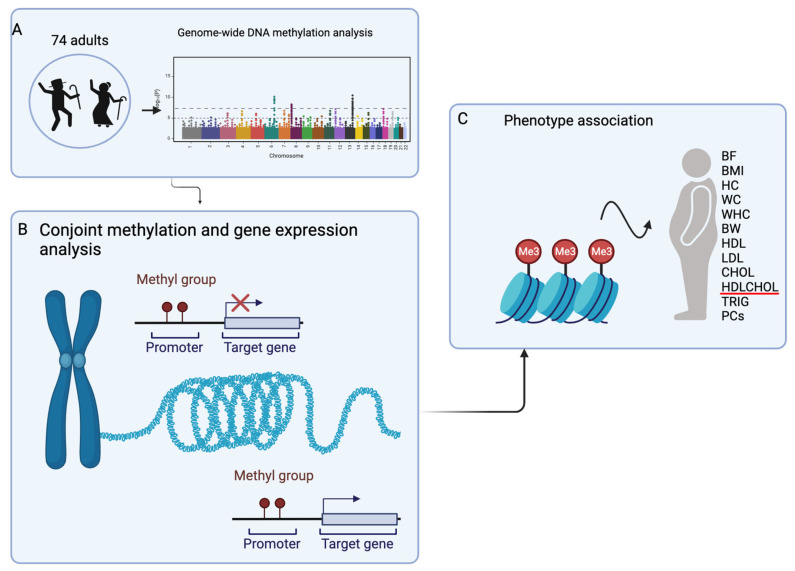
The summary of the workflow. (**A**) Genome-wide DNA methylation analysis with age. (**B**) The conjoint analysis of identified methylation and gene expression pairs. (**C**) The association of identified methylation CpGs in B to phenotypes. BF: body fat, P percentage, BMI: body mass index, HC: hip circumference, WC: waist circumference, WHR: waist-to-hip ratio, BW: body weight, HDL: high-density lipoprotein, LDL: low-density lipoprotein, CHOL: cholesterol, HDLCHOL: HDL to CHOL ratio, TRIGs: triglycerides, and PCs: principal components. Red line = example of blood lipid phenotype.

The summary of the cohort and workflow is displayed in Figure 1.

### 2.1. Sample and Phenotypic Characteristics

Through the Norfolk Island Health Study, participants volunteered to participate and completed the consent forms approved initially by the Griffith University Human Research Ethics Committee (HREC) and subsequently by the QUT HREC (no. 1600000464). Quantitative phenotypic data were collected at the same time as blood donation, including multiple obesity-related phenotypes. Body fat phenotypes included body fat percentage (BF, %, measured by infra-red refractance), body weight (BW, kg), body mass index (BMI, calculated as body weight divided by height squared in m^2^), waist circumference (cm), hip circumference (cm) and waist-to-hip ratio. Moreover, low- and high-density lipoprotein (LDL and HDL), total cholesterol (CHOL), triglyceride (TG), and the ratio of HDL to CHOL (HDLCHOL) were included in the analysis. The HDL, LDL, CHOL, and TG levels were measured in millimoles per litre (mmol/L). The details of these phenotypes can be found in a previously published study [25].

### 2.2. Principal Component Analysis

Missing values presented in the phenotypic variables were imputed by applying a parametric bootstrapping method in the missMDA package (version 1.14) [26]. Then, Principal Component Analysis (PCA) was performed using FactoMineR package (version 1.41) [27] to capture the correlation between body fat and lipid variables separately. This resulted in adding four principal components for body fat and lipid phenotypes, named BF.PC1, BF.PC2, LP.PC1, and LP.PC2, with the eigenvalue greater than 1. Each described measurement and PCs were treated as input phenotypes in the latter analysis.

### 2.3. Genomic Data

The extraction and purification of blood samples and DNA methylation analysis via Illumina methylation 450 K arrays have previously been described in Benton MC et al. [28]. DNA methylation raw data were obtained, and the methylation matrix quality control was performed in R through the ChAMP package (release 3.17) via the “champ.filter” function without removing CpGs annotated to contain SNPs [29]. Then, 433,525 CpGs remained for further analysis, and CpGs were annotated using the CpG feature profiles provided in the ChAMP package.

Gene expressions for corresponding samples were obtained from Illumina HumanHT-12 v.4 Expression BeadChip Kit arrays as previously described in Benton MC et al. [30]. There were 23,323 transcripts included after the log2 transformation and quantile normalisation. Genes were annotated using the illuminaHumanv4.db package (version 1.26.0).

### 2.4. Statistical Analysis

#### 2.4.1. Identification of Methylation Sites Associated with Age

A simple linear regression was performed with age as the outcome variable and CpG methylation as the independent variable to identify methylation sites associated with age. Significant CpGs were filtered based on(rle the genome-wide threshold of 2.4 × 10^−7^ [31].

#### 2.4.2. Conjoint Analysis of Methylation and Gene Expression Associated with Age

For genes with CpGs significantly associated with age, pairs of gene expression and methylation CpGs were used in the conjoint analysis. A pair of expression and methylation was selected when the methylation CpG mapped to the same gene as a gene expression CpG (i.e., was in cis). Therefore, each CpG for gene expression may be paired with multiple methylation CpGs. Using these, we tested for the interaction between methylation on gene expression to indicate age-related CpGs that act via gene expression. For this model, age was included as the outcome, while methylation, transcript, and its interaction term (methylation × transcript) were the independent variables. Significant pairs of methylation and transcript were selected if the *p*-value of CpGs, transcript, and interaction term were all less than 0.05. If there was more than one pair for the same gene, the one with the largest R^2^ was selected as the representative (index CpG).

#### 2.4.3. Association of Functional Age-Related CpGs on Body Fat Phenotypes

A series of linear regressions was performed where significant CpGs from the above conjoint analysis were considered independent variables and phenotypes as the dependent variables. This analysis adjusted the phenotype according to age, sex, and kinship. Kinship was the score of relatedness between individuals ranging from 0 to 1. The kinship score was included due to the potential confounder of family relationships between members in the cohort [32]. Sex was included as a covariate, given the significant difference in most phenotypes by sex, as seen in Table 1. The association was considered significant if the *p*-value was less than 0.05.

Due to the small sample size, the method used in conjoint analysis and functional age-related CpGs did not account for multiple testing to capture the preliminary result. All statistical analyses were performed in R (version 4.1.1) [33] and RStudio (version 1.4.1717) [34].

## 3. Results

### 3.1. Cohort Characteristics

The descriptive statistics of our cohort are summarised in Table 1. The cohort consisted of 24 females and 50 males. The mean age was 51.9 years, ranging from 31 to 83 years. The phenotypes included six anthropomorphic measurements for body fat compositions, summarised in Table 1. All of these exhibited significant differences between male and female subjects. Of five measured lipid phenotypes, only high-density lipoprotein (HDL) and HDL to cholesterol ratio (HDLCHOL) showed significant differences between males and females.

### 3.2. Epigenome-Wide Association Study of Age in NI Cohort

**Figure 2:**
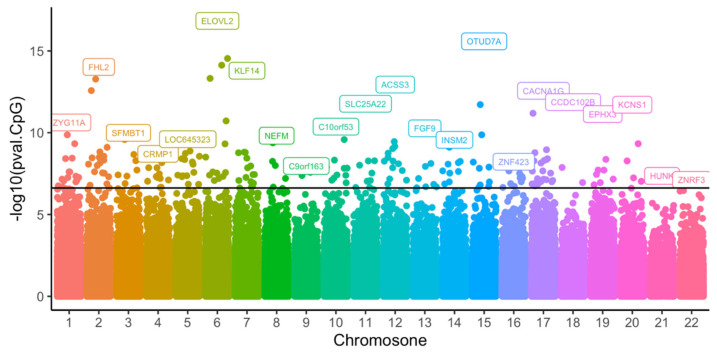
Epigenome-wide association of age in Norfolk Island cohort identified 226 CpGs annotated to 183 genes. Gene names shown in boxes indicate the most significant genes in each chromosome. The dark line indicates the significant threshold (*p* < 2.4 × 10^−7^).

**Figure 3:**
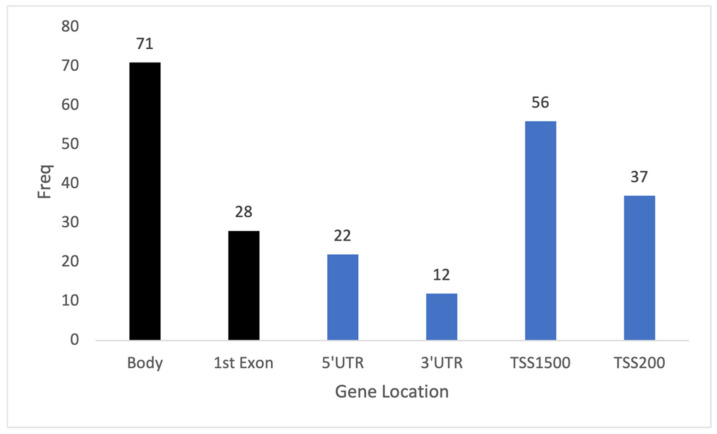
The distribution of 226 significant methylation sites within genes associated with age. The *x*-axis showed the list of gene locations while the *y*-axis showed the frequency of significant genes identified based on their positions. Black bars are non-regulatory regions. Blue bars are regulatory regions.

After the initial quality control of methylation data from the 74 blood samples, 433,525 CpGs remained for analysis. The association testing of CpG methylation with age was performed by applying a per-CpG linear regression. This identified 226 CpGs across 183 genes with *p*-values less than the epigenome-wide threshold (Figure 2). Of these 226 CpGs, more than half were located in gene regulatory regions (Figure 3). The top 10 CpGs ranked by R^2^ are summarized in Table 2. All CpGs, ranked by significance level and chromosome position, are shown in Supplementary Table S1.

The EWAS atlas was built to summarise findings from all EWAS studies and includes many studies aimed at age as the dependent variable. Filtering for age-associated CpGs in blood samples returned a list of 21,082 CpGs. Compared to the public gene list found in the EWAS atlas, there was an overlap of 164 CpGs annotated to 136 genes in the NI cohort.

### 3.3. Identification of Age-Related Expression CpGs

The aim of this study was to explore the functional mechanisms of age-related CpGs by linking these with gene expression levels within the same study subjects. We tested for age-related expression CpGs (eCpGs) by performing statistical interaction modelling. This modelling structure was used to identify CpGs that are associated with age in a manner dependent on gene expression (i.e., an interaction effect). Intersecting the measured gene sets derived from the methylation and expression arrays showed that 77 of the 183 age-related genes identified had data for both DNA methylation and gene expression. These CpGs and transcripts were included in the statistical models. This analysis resulted in the identification of 35 genes that showed evidence of an interaction effect between methylation on gene expression (i.e., eCpGs) in relation to aging (Table 3). Supplementary Table S2 displays the complete summaries of regression results, highlighting the highest R^2^ within each gene, as shown in the index column.

To further investigate whether the 35 genes identified were enriched in any specific biological processes, they were submitted to the ToppGene webpage [35]. Results of this analysis indicted two significant Gene Ontology hits involving molecular function on DNA-binding transcription factor activity, namely GO:0000981 (p_bonferroni_ = 0.032, 10 genes) and GO:0003700 (p_bonferroni_ = 0.044, 10 genes).

### 3.4. Age-Related eCpGs and Body Fat Phenotypes

The identified 35 age-related eCpGs were followed up to explore associations in relation to obesity-related phenotypes. To do this, a series of linear regressions was performed and adjusted by sex, age, and kinship. Table 4 summarizes the significant CpGs associated with phenotypes using the CpGs lists highlighted in Table 3. CpGs with a *p*-value less than 0.05 were considered significant. As a result, ten genes contained CpGs for which methylation levels were associated with one or more phenotypes, with R^2^ ranging from 0.2 to 0.53. Specifically, there were four genes with methylation located on the gene body and six genes with methylated CpGs found in surrounding regulatory regions. The top four genes with the highest R^2^ were Cytoplasmic Polyadenylation Element Binding Protein 2 (*CPEB2*), TIA1 cytotoxic granule associated RNA binding protein like 1 (*TIAL1*), Complement C1s (*C1S*), and *ANKRD11*. Among these, cg05638739, located on 5′UTR of the *ANKRD11*, was associated with nine out of fifteen tested phenotypes. Most of the associations between cg05638739 and phenotypes were positive, except for the negative associations with HDL.

### 3.5. Exploring Causal Links of eCpGs with Adiposity

**Figure 4:**
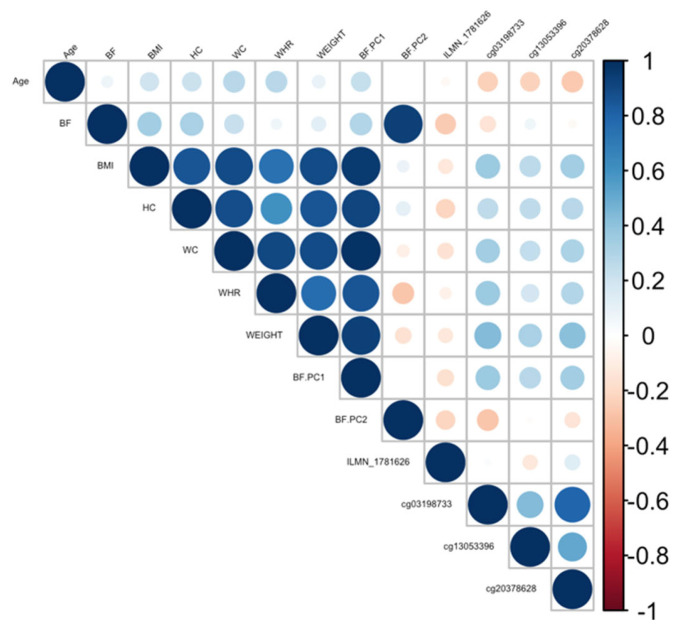
Correlation plot between all CpGs, transcripts, body fat phenotypes, and age generated for the *C1S* gene. BF: body fat percentage, BMI: body mass index, HC: hip circumference, WC: waist circumference, WHR: waist-to-hip ratio, Weight: body weight, BF.PC1: principal component 1, BF.PC2: principal component 2.

**Figure 5:**
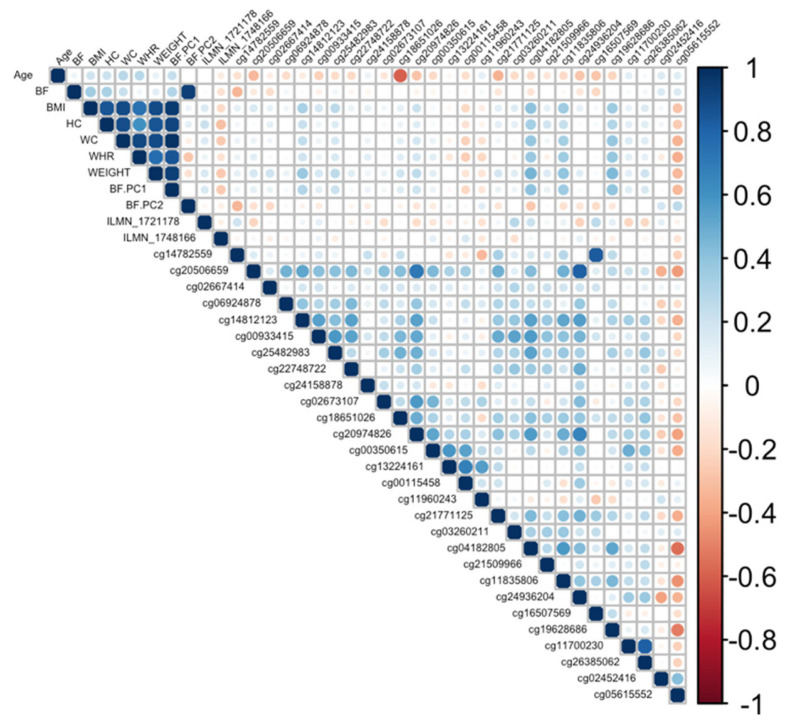
Correlation plot between all CpGs, transcripts, body fat phenotypes, and age generated for the *COL11A2* gene. BF: body fat percentage, BMI: body mass index, HC: hip circumference, WC: waist circumference, WHR: waist-to-hip ratio, Weight: body weight, BF.PC1: principal component 1, BF.PC2: principal component 2.

**Figure 6:**
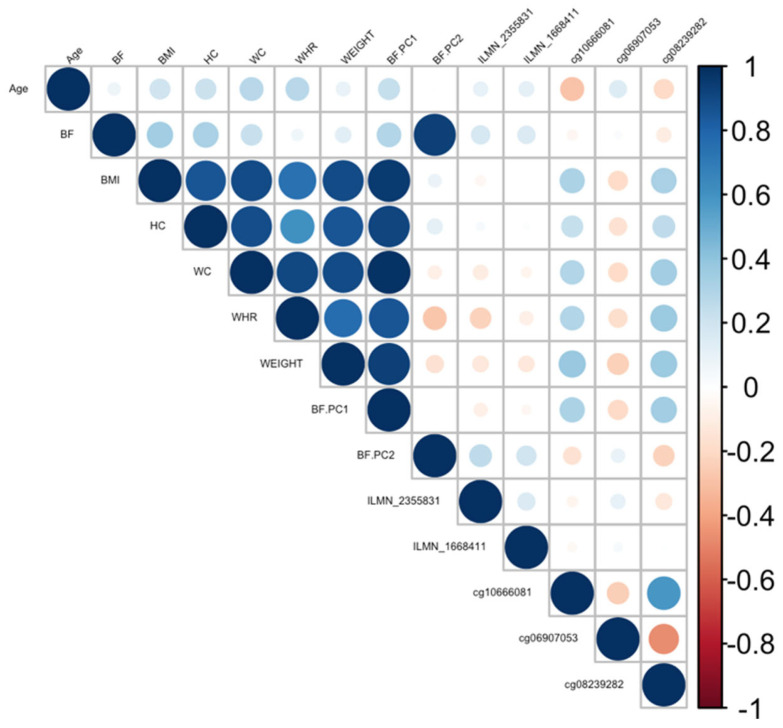
Correlation plot between all CpGs, transcripts, body fat phenotypes and age generated for the FHL2 gene. BF: body fat percentage, BMI: body mass index, HC: hip circumference, WC: waist circumference, WHR: waist-to-hip ratio, Weight: body weight, BF.PC1: principal component 1, BF.PC2: principal component 2.

Ranking genes by R^2^ may only be partially useful, as some genes will be *indirectly* associated with body fat phenotypes due to age association and not functional causality. To best interpret the functional (causal) importance of the top 10 obesity-related genes, a critical and systematic method was required. Notably, our examined transcripts were derived from blood cells, not adipose tissue. Thus, we ideally required a link to adipose tissue for the genes to be functionally causal. To this end, a literature-based assessment of the functional role of age-related CpGs in adipose tissue and obesity was performed using the terms “gene AND obesity” and “gene AND adipose” for all 10 genes. Priority was then assigned to genes based on whether any published studies showed evidence of functional involvement (Supplementary Table S3). This assessment showed that only three out of the 10 genes provided good functional evidence for involvement in adiposity. Ranked by max R^2,^ these were *C1S*, *COL11A2*, Four and *FHL2*. Specifically, these genes are epigenetically and transcriptionally variable in adipose tissue among subjects with obesity and/or metabolic syndrome [36,37,38,39]. To visually assess the DNA methylation patterns in relation to gene expression, body fat, and age, a correlation plot between all CpGs, transcripts, body fat phenotypes, and age was generated for each gene (Figure 4, Figure 5 and Figure 6).

## 4. Discussion

Here, we investigated functional epigenetic sites associated with age- and obesity-related phenotypes in the NI cohort. We identified 226 significant CpG CpGs associated with age annotated to 183 genes; 164 of the CpG CpGs overlapping with the Aging EWAS Atlas at 77 of the 183 genes had both DNA methylation and gene expression profiles, from which 35 genes showed a mediation effect between methylation and gene expression.

Of 35 indexed CpGs highlighted in Table 3, only two CpGs were presented in the 226 significant CpGs to be associated with age, indicating that these CpGs directly affected gene expression on the same gene in which the CpGs were located. Interestingly, the remaining CpGs only showed an association with age on the same gene after the addition of gene expressions and the interaction of these factors. This might suggest that these CpGs act as mediators to age rather than having a direct effect. Moreover, many of the significant CpGs associated with age were eliminated after adding gene expressions and their interactions. The potential reason for this elimination could be due to the insignificance of transcription or the interaction term in the regression model. Another reason could be the small sample size, potentially decreasing statistical power.

In our study, *COL11A2* was the most significant gene, with 28 CpGs associated with both age and gene expression. In particular, CpG cg18651026 was associated with three phenotypes: WC, WHR, and BF.PC1. The *COL11A2* is located on chromosome 6, and encodes one of two alpha chains of type XI collagen, a fibrillary collagen found in multiple tissues across the body, such as the cartilage, tendons, trachea, etc. The disruption of type XI collagen loosens extracellular matrix (ECM) connections by disrupting the correct fibril assembly [40]. Mutations in the *COL11A2* gene cause skeletal abnormalities [41]. Interestingly, *COL11A2* has also been implicated as an insulin-resistant gene epigenetically regulated in visceral adipose tissue in a morbidly obese cohort [37]. Furthermore, in an investigation of SNPs associated with the methylation of *COL11A2* genes, two identified rare alleles of these methylation sites showed an association with plasma fasting glucose levels in an obese cohort of metabolic syndrome [36]. However, methylation of the *COL11A2* sites was not associated with body fat phenotypes. The direct relationship between the *COL11A2* gene and WC remains unclear. The waist is comprised of many adipose cells, which typically increase with age. Therefore, the enlargement of these tissues requires a remodelling of the ECM [42]. Collagen is involved in correct fibril assembly and the correct formation of some of the other components in the ECM to ensure a healthy function of adipose tissues and insulin sensitivity. Further functional analysis of the *COL11A2* gene would be beneficial to understand the impact of collagen on the ECM and other connective tissues and a potential relationship with body fat phenotypes.

Another gene found to be associated with five body fat phenotypes (BF, BMI, HC, WC, and BF.PC1) was the *C1S* gene. The *C1S* gene belongs to a C1 complex including C1q, C1r, and C1s subunit. This is part of the classical pathway, in which the activation of C1s triggers the formation of a membrane complex to promote inflammation and autoimmunity. The function of C1s was discussed in a previous review by Ye et al. [43]; however, a direct role for C1s in body fat phenotypes has not previously been described. It is known that C1q binding to antigen–antibody immune complexes will activate C1s and the C1 complex, and that C1q can activate the WNT signalling pathway that is known to play a role in obesity [44]. In addition, the upregulation of C1q was observed in adipose tissue in the ob/ob mice [45]. Furthermore, a study on rare monozygotic twin pairs showed the upregulation of the *C1s* gene in adipose tissue in heavier BMI co-twins and obese males, suggesting the increased inflammatory activity of complement pathway components [38]. Our study showed a negative association between DNA methylation and gene expression and a negative association between DNA methylation and body fat phenotypes. The methylation CpGs are situated on the promoter of the *C1S* gene and therefore potentially control the expression of *C1S* during the activation of the immune system.

In our study, the methylation of the *FHL2* gene was positively associated with BF. Both methylation and the expression of the *FHL2* gene in the blood and adipose tissue increase with age [39]. Interestingly, hyper-methylation of *FHL2* increases its expression [39]. A previous study reported higher expressions of the *FHL2* gene in the white adipose tissue when comparing obese and lean individuals [46]. They also showed in mice that a decrease in *FHL2* could be beneficial in in preventing weight gain when exposed to a high-fat diet. In addition, the *FHL2* gene affects the formation of adipocytes [47]. Altogether, these data support our findings, which suggest the association of *FHL2* and BF.

As well as the significantly associated DMR on *COL11A2*, a large group of 116 CpGs annotated to the *ANKRD11* gene on chromosome 16 were associated with age after adding mediation terms. The index CpG of this gene was cg05638739, which was associated with nine out of fifteen tested phenotypes. Notably, this CpG was positively associated with seven phenotypes in the body fat sub-group, except for WHR. It was also associated with HDL and HDLCHOL. Increased *ANKRD11* expression elevated the expression of the tumour suppressor p53 to protect normal cells against cell cycle dysregulation. In addition to suppressing tumour growth, recent reviews suggest a vital role of p53 in adipogenesis [48] and glucose metabolism [49], both processes crucial to the development of obesity. In our study, the index CpG (cg05638739) was located on the 5′UTR of the *ANKRD11* gene, which may result in enhanced p53 gene transcription, suggesting that *ANKRD11* may play a role in controlling fat cells by activating p53 pathways.

The *CPEB2* gene was associated with six phenotypes within the body fat subgroup, except for WHR and WC. *CPEB2* is a protein-coding gene belonging to the *CPEB* family, which act as transcription regulators. While other genes within the *CPEB* family, including *CPEB1*, *CPEB3*, and *CPEB4*, have been associated with aging, a role for *CPEB2* has not been previously reported. The interaction of CPEB2 with eukaryotic elongation factor 2 (eEF2) regulates the translation of hypoxia-inducible factor 1α (HIF1A) mRNA, which has been proven to increase in adipose tissue in obese mice [50,51]. In addition, *CPEB2* expression is upregulated in renal cancer cell proliferation and migration via the inactivation of tumour suppressor p53 [52]. In obesity research, the knockout of the *CPEB2* gene showed its ability to reduce thermogenesis in brown adipose tissue [53].

Interestingly, the *TIAL1* gene was positively associated with all body fat subgroups. TIAL1 is TIA-1-related/like protein. Research has shown that TIAL1 acts as a cellular sensor; knocking out this protein promoted the expression of many mRNAs in cell proliferation and apoptosis [54,55,56,57]. However, our study is the first to show a positive association of the *TIAL1* gene with multiple body fat phenotypes. How this gene is involved in the control of body fat cells is not clear; however, we suggest that *TIAL1* expression may control cell proliferation and apoptosis to maintain a healthy balance of adipose cells.

Our study analysed genomic data from an isolated healthy cohort of 74 individuals. Due to the small sample size, the statistical method did not address multiple testing in model 2 and model 3, as it was an exploratory study. Furthermore, this study only considered genes that have been found to be associated with age in model 1 to model 2, resulting in 77 out of 183 genes with both gene expression and methylation data. An independent and larger general population would be required to validate these results. In addition, a larger sample size would help investigate additional covariates such as cell proportions and SNP genotypes on these associations.

In conclusion, there were 35 age-related expression CpGs. Among these eCpGs, ten genes were associated with at least one obesity-related phenotype. While the function of these genes’ impact on body fat metabolism is unclear, these genes were shown to participate in multiple related biological processes, including the arrangement of ECM and cell proliferation/cell cycles as well as the immune system. While promising targets for future studies, these new findings still need further validation and functional evaluation to clarify their role in aging and obesity.

## Figures and Tables

**Table 1:** Summary of body fat and lipid phenotypes in NI cohort.

	Female (N = 24)	Male (N = 50)	Total (N = 74)	*p*
Age	51 (12.8)	52.3 (12.1)	51.9 (12.2)	0.6
Body Fat Percentage (%)	34.1 (5.3)	25.4 (7.8)	28.2 (8.1)	<0.001 *
Body Mass Index (kg/m^2^)	23.3 (2.3)	27.9 (4.3)	26.4 (4.3)	<0.001 *
Hip Circumference (cm)	98.2 (6.1)	105.9 (8.1)	103.4 (8.3)	<0.001 *
Waist Circumference (cm)	81.3 (9.4)	99.1 (12.8)	93.3 (14.4)	<0.001 *
Waist-to-Hip Ratio	0.8 (0.06)	0.9 (0.1)	0.9 (0.1)	<0.001 *
Body Weight (kg)	63.3 (7.67)	89.36 (14.6)	81 (17.7)	<0.001 *
Total Cholesterol (mmol/L)	5.6 (0.85)	5.71 (1.01)	5.7 (0.96)	0.586
HDL-to-CHOL ratio (HDLCHOL)	3.4 (0.74)	4.53 (1.57)	4.15 (1.46)	<0.001 *
Low Density Lipoprotein (mmol/L)	3.4 (0.77)	3.68 (0.98)	3.6 (0.9)	0.24
High Density Lipoprotein (mmol/L)	1.7 (0.33)	1.37 (0.4)	1.48 (0.41)	<0.001 *
Triglycerides (mmol/L)	1.01 (0.35)	1.68 (1.7)	1.47 (1.45)	0.063

Data were summarized as mean (standard deviation). * statistically significant.

**Table 2:** The summary statistics of the top 10 significant CpGs associated with age (*p* < 2.4 × 10^−7^).

CpG	Beta	*p*	R^2^	CHR	MAPINFO	Gene	Feature
cg16867657	0.8	1.4 × 10^−17^	0.64	6	11044877	*ELOVL2*	TSS1500
cg04875128	0.78	2.6 × 10^−16^	0.61	15	31775895	*OTUD7A*	Body
cg22736354	0.76	2.9 × 10^−15^	0.58	6	18122719	*NHLRC1*	1stExon
cg22454769	0.75	1.1 × 10^−14^	0.57	2	106015767	*FHL2*	TSS200
cg24079702	0.75	9.5 × 10^−15^	0.57	2	106015771	*FHL2*	TSS200
cg24724428	0.76	7.4 × 10^−15^	0.57	6	11044888	*ELOVL2*	TSS1500
cg14361627	0.75	1.6 × 10^−14^	0.56	7	130419116	*KLF14*	TSS1500
cg23606718	0.74	5.3 × 10^−14^	0.55	2	131513927	*FAM123C*	5′UTR
cg21572722	0.74	4.7 × 10^−14^	0.55	6	11044894	*ELOVL2*	TSS1500
cg11649376	−0.73	1.1 × 10^−13^	0.54	12	81473234	*ACSS3*	Body

All of these 10 genes were associated with age in previous studies. Beta: standardised regression coefficients.

**Table 3:** Interactive effect of methylation and gene expression on relation to age.

CpG	Transcript	CpG		Transcript		CpG × Transcript		R^2^	CHR	Position	Gene	Feature	R ^a^	Known ^b^
		Beta	*p*	Beta	*p*	Beta	*p*							
cg18651026	ILMN_1748166	5.35	4.30 × 10^−2^	3.16	2.90 × 10^−2^	−6.87	2.50 × 10^−2^	0.4	6	33140660	*COL11A2*	Body	0.04	1
cg00743094	ILMN_1663538	−3.09	4.30 × 10^−2^	−1.21	7.30 × 10^−3^	3.9	1.70 × 10^−2^	0.39	13	100547968	*CLYBL*	3′UTR	0.09	0
cg11872672	ILMN_1728844	−7.36	2.60 × 10^−2^	−5.7	3.70 × 10^−2^	7.75	3.90 × 10^−2^	0.36	7	157514730	*PTPRN2*	Body	−0.23	0
cg13096208	ILMN_1855910	9.05	4.60 × 10^−3^	1.49	3.10 × 10^−3^	−8.63	6.40 × 10^−3^	0.24	18	55019843	*ST8SIA3*	1stExon	−0.11	0
cg05638739	ILMN_1690465	−10.44	4.40× 10^−5^	−12.94	5.40× 10^−5^	18	5.30× 10^−5^	0.22	16	89440324	*ANKRD11*	5′UTR	0.19	0
cg23095192	ILMN_1820767	−8.89	1.90 × 10^−2^	−3.94	3.20 × 10^−2^	8.89	2.20 × 10^−2^	0.2	2	145271307	*ZEB2*	Body	−0.19	0
cg20332195	ILMN_1682449	−10.72	9.20 × 10^−3^	−2.15	9.00 × 10^−3^	11.19	7.30 × 10^−3^	0.2	4	10459929	*ZNF518B*	TSS1500	−0.04	0
cg06799422	ILMN_2372379	7.08	1.70 × 10^−2^	1.54	1.70 × 10^−2^	−7	2.30 × 10^−2^	0.19	15	41952235	*MGA*	TSS1500	0.07	1
cg22088743	ILMN_2133675	9.56	4.70 × 10^−3^	16.9	3.50 × 10^−3^	−18.97	3.70 × 10^−3^	0.19	17	78183317	*SGSH*	3′UTR	−0.06	0
cg10666081	ILMN_2355831	−6.92	8.40 × 10^−3^	−12.79	1.20 × 10^−2^	14.17	1.10 × 10^−2^	0.17	2	105985002	*FHL2*	Body	−0.05	0
cg17761990	ILMN_2391750	8.32	1.40 × 10^−3^	14.69	1.00 × 10^−3^	−17.13	1.10 × 10^−3^	0.17	3	53042940	*SFMBT1*	5′UTR	0.03	0
cg07198402	ILMN_1749667	8.69	2.40 × 10^−2^	10.48	2.00 × 10^−2^	−14.3	2.00 × 10^−2^	0.17	1	228395145	*OBSCN*	TSS1500	0.07	0
cg14082919	ILMN_2352295	11.16	8.20 × 10^−3^	14.14	7.30 × 10^−3^	−16.69	6.80 × 10^−3^	0.17	11	129814820	*PRDM10*	Body	−0.17	0
cg18443378	ILMN_1746552	−5.65	4.20 × 10^−2^	−1.52	3.10 × 10^−2^	6.09	3.20 × 10^−2^	0.16	4	176986950	*WDR17*	TSS200	−0.04	0
cg04662983	ILMN_1708508	−9.33	6.80 × 10^−3^	−1.88	3.20 × 10^−3^	9.43	5.80 × 10^−3^	0.16	17	56834321	*PPM1E*	Body	−0.14	0
cg20671534	ILMN_1658576	−6.04	6.50 × 10^−3^	−2.56	1.20 × 10^−2^	6.65	8.80 × 10^−3^	0.15	2	220174629	*PTPRN*	TSS1500	0.12	0
cg13053396	ILMN_1781626	−10.19	4.50 × 10^−3^	−8.53	5.10 × 10^−3^	12.23	5.40 × 10^−3^	0.15	12	7168545	*C1S*	5′UTR	−0.13	0
cg16765387	ILMN_1719975	−5.3	7.10 × 10^−4^	−3.14	8.30 × 10^−4^	5.9	7.20 × 10^−4^	0.15	12	54411245	*HOXC4*	5′UTR	−0.09	0
cg19929852	ILMN_1774948	9.08	1.00 × 10^−2^	13.15	8.60 × 10^−3^	−15.21	9.60 × 10^−3^	0.14	4	15068643	*CPEB2*	3′UTR	−0.08	0
cg02764478	ILMN_1696279	6.66	3.70 × 10^−2^	1.85	4.90 × 10^−2^	−6.46	4.50 × 10^−2^	0.14	6	100904316	*SIM1*	Body	−0.1	1
cg19262958	ILMN_1687958	4.49	4.80 × 10^−2^	8.21	4.20 × 10^−2^	−9.57	3.80 × 10^−2^	0.14	11	792861	*SLC25A22*	Body	−0.02	0
cg26365090	ILMN_2082209	−10.07	1.20 × 10^−2^	−0.55	2.70 × 10^−3^	10.11	1.30 × 10^−2^	0.13	20	42574362	*TOX2*	5′UTR	0.21	0
cg13539203	ILMN_1768483	9.55	7.80 × 10^−3^	10.02	7.80 × 10^−3^	−14.08	7.10 × 10^−3^	0.12	2	26950545	*KCNK3*	Body	0.01	0
cg08858926	ILMN_1808587	−7.5	8.90 × 10^−3^	−3.74	1.20 × 10^−2^	7.97	1.00 × 10^−2^	0.12	16	72918832	*ZFHX3*	Body	−0.09	0
cg22743761	ILMN_1739366	−5.7	3.20 × 10^−2^	−2.22	2.70 × 10^−2^	6.85	2.60 × 10^−2^	0.11	2	162273648	*TBR1*	Body	0.26	0
cg17949162	ILMN_1796855	13.11	1.60 × 10^−2^	1.51	9.10 × 10^−3^	−13.17	1.70 × 10^−2^	0.11	10	121355153	*TIAL1*	Body	0.03	0
cg04256697	ILMN_1684440	5.15	1.30 × 10^−2^	3.05	8.50 × 10^−3^	−6.22	1.20 × 10^−2^	0.11	12	120688557	*PXN*	Body	0.1	0
cg10912240	ILMN_2149946	−5.43	3.20 × 10^−2^	−1.76	2.40 × 10^−2^	5.99	2.60 × 10^−2^	0.11	14	29235907	*FOXG1*	TSS1500	0.06	1
cg00866814	ILMN_1668194	−5.78	7.00 × 10^−3^	−2.98	9.10 × 10^−3^	6.46	8.40 × 10^−3^	0.11	19	49017364	*LMTK3*	TSS1500	0.03	0
cg09628707	ILMN_1761903	7.53	8.70 × 10^−3^	3.09	9.00 × 10^−3^	−7.86	9.70 × 10^−3^	0.11	20	43729410	*KCNS1*	5′UTR	−0.05	0
cg17370322	ILMN_1788457	7.85	1.40 × 10^−2^	0.88	7.80 × 10^−3^	−7.95	1.50 × 10^−2^	0.1	13	95953482	*ABCC4*	Body	0.16	0
cg03578473	ILMN_1669425	−9.72	1.30 × 10^−2^	−1.15	1.30 × 10^−2^	9.9	1.20 × 10^−2^	0.09	2	182546504	*NEUROD1*	TSS1500	0.05	0
cg22509041	ILMN_2119692	5.42	4.10 × 10^−2^	1.18	2.40 × 10^−2^	−5.6	4.30 × 10^−2^	0.09	12	53574524	*CSAD*	TSS200	0.15	0
cg11718501	ILMN_1666310	5.18	4.20 × 10^−2^	1.13	2.30 × 10^−2^	−5.09	4.10 × 10^−2^	0.08	11	122850972	*BSX*	Body	−0.21	0
cg08198377	ILMN_1691181	−3.6	2.70 × 10^−2^	−1.84	3.20 × 10^−2^	3.62	2.80 × 10^−2^	0.07	14	51707975	*TMX1*	Body	−0.24	0

^a^: Correlation value between methylation and gene expression. ^b^: Known age-related CpG (no = 0; yes = 1). According to largest effect size (R^2^), the Collagen Type XI Alpha 2 Chain (*COL11A2*) gene located on the gene body of chromosome 6 contained a functional eCpG associated with age. The following four most significant genes were Citramalyl-CoA Lyase (*CLYBL*), Protein Tyrosine Phosphatase Receptor Type N2 (*PTPRN2*), ST8 Alpha-N-Acetyl-Neuraminide Alpha-2,8-Sialyltransferase 3 (*ST8SIA3*), and Ankyrin Repeat Domain Containing 11 (*ANKRD11*), located on chromosomes 13, 7, 18, and 16, respectively. Except for *ST8SIA3*, which had a sole CpG associated with age, the other four genes had more than 5 CpGs associated with age through the interaction with gene expression, forming functional differentially methylated regions (DMRs) related to age.

**Table 4:** Age-related eCpGs associated with body fat phenotypes.

Phenotype	CpG	CHR	MAPINFO	Gene	Feature	Beta	*p*	R^2^
BF	cg10666081	2	105985002	*FHL2*	Body	0.25	3.5 × 10^−2^	0.31
HC	cg20671534	2	220174629	*PTPRN*	TSS1500	0.25	2.4 × 10^−2^	0.29
BF	cg19929852	4	15068643	*CPEB2*	3′UTR	−0.3	3.6 × 10^−3^	0.35
BMI						−0.27	9.9 × 10^−3^	0.35
BW						−0.21	1.5 × 10^−2^	0.53
HC						−0.24	2.6 × 10^−2^	0.29
BF.PC1						−0.23	1.8 × 10^−2^	0.41
BF.PC2						−0.26	2.7 × 10^−3^	0.56
WC	cg18651026	6	33140660	*COL11A2*	Body	0.28	1.9 × 10^−2^	0.44
WHR						0.28	1.3 × 10^−2^	0.50
BF.PC1						0.26	3.5 × 10^−2^	0.40
BF	cg17949162	10	121355153	*TIAL1*	Body	0.41	6.1 × 10^−4^	0.38
BMI						0.45	1.0 × 10^−4^	0.43
BW						0.29	3.9 × 10^−3^	0.55
HC						0.32	9.2 × 10^−3^	0.30
WC						0.39	2.7 × 10^−4^	0.50
WHR						0.35	6.8 × 10^−4^	0.54
BF.PC1						0.41	2.1 × 10^−4^	0.47
BF.PC2						0.28	5.1 × 10^−3^	0.55
BF	cg11718501	11	122850972	*BSX*	Body	−0.29	2.3 × 10^−2^	0.32
WHR						−0.23	4.2 × 10^−2^	0.48
BF.PC2						−0.21	4.9 × 10^−2^	0.53
BMI	cg13053396	12	7168545	*C1S*	5′UTR	0.24	2.1 × 10^−2^	0.34
BW						0.24	6.3 × 10^−3^	0.54
HC						0.25	2.2 × 10^−2^	0.29
WC						0.22	2.2 × 10^−2^	0.44
BF.PC1						0.25	1.3 × 10^−2^	0.41
BF	cg05638739	16	89440324	*ANKRD11*	5′UTR	0.27	1.3 × 10^−2^	0.33
BMI						0.34	1.7 × 10^−3^	0.38
BW						0.21	2.4 × 10^−2^	0.52
HC						0.25	3.0 × 10^−2^	0.28
WC						0.24	1.8 × 10^−2^	0.45
HDL						−0.23	4.9 × 10^−2^	0.20
CHOLHDL						0.24	4.6 × 10^−2^	0.22
BF.PC1						0.27	7.7 × 10^−3^	0.42
BF.PC2						0.21	2.2 × 10^−2^	0.53
BMI	cg26365090	20	42574362	*TOX2*	5′UTR	−0.21	4.1 × 10^−2^	0.33
BW	cg09628707	20	43729410	*KCNS1*	5′UTR	0.18	3.9 × 10^−2^	0.52

## Data Availability

Data will be shared with researchers upon request and on a collaborative basis.

## Supplementary Materials

The following supporting information can be downloaded at: https://www.mdpi.com/article/10.3390/cimb45100497/s1, Table S1: The summary statistic of significant probes associated with age (*p* < 2.4 × 10^−7^) ordered by Chromosome and position; Table S2: Age-related eCpGs; Table S3: Literature-based assessment of the functional role of age-related eCpG genes in adipose tissue and obesity.
