# Supplementary material for: Exploring the Functional Basis of Epigenetic Aging in Relation to Body Fat Phenotypes in the Norfolk Island Cohort
Source: Curr Issues Mol Biol. 2023 Sep 27;45(10):7862–77. doi: 10.3390/cimb45100497 (PMC10605526; doi:10.3390/cimb45100497)
Supplement: Supplementary file 1 [file cimb-45-00497-s001.zip › cimb-2585859-supplementary.pdf]

Supplementary Table S1 The summary statistic of significant probes associated with age ($P < 2.4 \times 10^{-7}$) ordered by Chromosome and position.

CpG	Beta	P	R^2	CHR	MAPINFO	Gene	feature	Known*
cg20822990	-0.58	4.6e-08	0.34	1	17338766	*ATP13A2*	TSS1500	1
cg09793172	0.58	7.0e-08	0.33	1	21044101	*KIF17*	1stExon	0
cg15728256	0.58	8.3e-08	0.33	1	40254184	*BMP8B*	5'UTR	0
cg18815943	0.62	3.6e-09	0.39	1	47882314	*FOXE3*	1stExon	1
cg01281911	0.57	9.0e-08	0.33	1	47882686	*FOXE3*	1stExon	1
cg06784991	0.68	2.4e-11	0.46	1	53308768	*ZYG11A*	Body	1
cg24466241	0.56	2.2e-07	0.31	1	53308908	*ZYG11A*	Body	1
cg10628205	-0.66	1.4e-10	0.44	1	61547131	*NFIA*	Body	1
cg12079303	-0.57	1.0e-07	0.33	1	61547163	*NFIA*	Body	0
cg23078123	-0.65	4.7e-10	0.42	1	68577796	*GPR177*	Body	1
cg20591472	0.62	3.9e-09	0.38	1	110008990	*SYPL2*	TSS200	1
cg01974375	-0.58	5.5e-08	0.34	1	151298954	*PI4KB*	TSS1500	1
cg03890680	-0.59	2.5e-08	0.35	1	227504721	*CDC42BPA*	1stExon	1
cg01557798	0.58	5.5e-08	0.34	1	228400628	*OBSCN*	Body	1
cg09809672	-0.56	1.8e-07	0.32	1	236557682	*EDARADD*	TSS1500	1
cg03422911	0.59	2.5e-08	0.35	1	237205295	*RYR2*	TSS1500	1
cg20491914	0.62	4.8e-09	0.38	2	26915349	*KCNK3*	TSS1500	0
cg27320127	0.61	7.0e-09	0.37	2	47798396	*KCNK12*	TSS1500	1
cg01968178	0.56	1.6e-07	0.32	2	86565038	*REEP1*	Body	1

cg06639320	0.73	2.7e-13	0.53	2	106015739	*FHL2*	TSS200	1
cg22454769	0.75	1.1e-14	0.57	2	106015767	*FHL2*	TSS200	1
cg24079702	0.75	9.5e-15	0.57	2	106015771	*FHL2*	TSS200	1
cg13381486	0.59	2.5e-08	0.35	2	131513632	*FAM123C*	TSS1500	1
cg23606718	0.74	5.3e-14	0.55	2	131513927	*FAM123C*	5'UTR	1
cg00573770	-0.61	8.4e-09	0.37	2	145278485	*ZEB2*	TSS1500	0
cg00602811	-0.59	2.9e-08	0.35	2	145278564	*ZEB2*	TSS1500	1
cg18311633	-0.58	5.0e-08	0.34	2	155309539	*GALNT13*	3'UTR	0
cg12757011	0.64	7.8e-10	0.41	2	162281111	*TBR1*	3'UTR	1
cg01620164	-0.62	2.8e-09	0.39	2	164590272	*FIGN*	Body	1
cg03767531	0.62	3.2e-09	0.39	2	182545572	*NEUROD1*	TSS200	0
cg03545227	0.63	1.5e-09	0.4	2	220173100	*PTPRN*	Body	1
cg23988310	0.59	3.0e-08	0.35	2	224467049	*SCG2*	5'UTR	0
cg12001304	0.61	1.0e-08	0.37	2	229045020	*SPHKAP*	Body	0
cg24436906	0.57	1.4e-07	0.32	2	242498081	*BOK*	TSS200	1
cg21117668	0.62	3.4e-09	0.39	2	242498349	*BOK*	5'UTR	1
cg22934295	0.62	5.2e-09	0.38	3	42306385	*CCK*	1stExon	0
cg12091542	0.57	1.3e-07	0.32	3	48700425	*CELSR3*	TSS200	0
cg16323609	-0.58	4.5e-08	0.34	3	48885591	*PRKAR2A*	TSS1500	0
cg01844642	0.65	2.6e-10	0.43	3	51989764	*GPR62*	1stExon	1
cg04474832	-0.6	1.9e-08	0.36	3	52008487	*ABHD14B*	1stExon	1
cg03607117	0.67	1.0e-10	0.44	3	53080440	*SFMBT1*	TSS1500	1
cg09379755	0.56	2.4e-07	0.31	3	62861925	*CADPS*	TSS1500	0

cg15618978	0.59	3.8e-08	0.34	3	160167990	*TRIM59*	TSS1500	1
cg00481951	0.63	2.1e-09	0.39	3	187387650	*SST*	Body	1
cg07963234	0.6	1.4e-08	0.36	4	5894923	*CRMP1*	TSS200	1
cg15448975	0.63	2.0e-09	0.4	4	5894930	*CRMP1*	TSS200	1
cg22680204	0.58	4.8e-08	0.34	4	5895098	*CRMP1*	TSS1500	0
cg21523251	0.6	1.4e-08	0.36	4	8582110	*GPR78*	TSS200	0
cg11970349	0.61	6.8e-09	0.37	4	8582287	*GPR78*	TSS200	1
cg23995914	0.58	5.6e-08	0.34	4	10459228	*ZNF518B*	TSS200	1
cg07809027	-0.56	2.2e-07	0.31	4	15007205	*CPEB2*	Body	0
cg18797590	-0.56	2.2e-07	0.31	4	15480643	*CC2D2A*	Body	0
cg20816447	-0.57	9.4e-08	0.33	4	15480781	*CC2D2A*	Body	1
cg13726191	-0.59	3.3e-08	0.35	4	15940033	*FGFBP1*	TSS200	1
cg24199834	0.58	6.3e-08	0.34	4	147560126	*POU4F2*	1stExon	1
cg01102833	-0.58	7.8e-08	0.33	4	176986621	*WDR17*	TSS1500	0
cg25302419	0.62	3.9e-09	0.38	5	11904015	*CTNND2*	5'UTR	0
cg06782035	0.62	4.4e-09	0.38	5	16179135	*MARCH11*	Body	1
cg23479922	0.57	1.4e-07	0.32	5	16179633	*MARCH11*	1stExon	1
cg09017434	0.59	4.3e-08	0.34	5	16179660	*MARCH11*	1stExon	1
cg12238343	0.58	4.9e-08	0.34	5	33936402	*RXFP3*	TSS200	1
cg17621438	-0.63	1.9e-09	0.4	5	63461216	*RNF180*	TSS1500	1
cg07850154	-0.63	1.3e-09	0.4	5	63461232	*RNF180*	TSS1500	1
cg23008153	-0.59	4.3e-08	0.34	5	63461305	*RNF180*	TSS1500	0
cg12563644	0.65	2.5e-10	0.43	5	87956996	*LOC645323*	Body	0

cg11700800	0.61	6.2e-09	0.38	5	92906633	*FLJ42709*	Body	0
cg02699218	0.62	2.8e-09	0.39	5	132150128	*ANKRD43*	1stExon	1
cg06448705	0.57	1.4e-07	0.32	5	135701094	*TRPC7*	5'UTR	0
cg21874213	-0.61	8.6e-09	0.37	5	139229292	*NRG2*	Body	1
cg13900817	-0.59	2.7e-08	0.35	5	172070153	*NEURL1B*	Body	0
cg06933824	0.59	4.0e-08	0.34	5	172110579	*NEURL1B*	Body	1
cg16867657	0.8	1.4e-17	0.64	6	11044877	*ELOVL2*	TSS1500	1
cg24724428	0.76	7.4e-15	0.57	6	11044888	*ELOVL2*	TSS1500	1
cg21572722	0.74	4.7e-14	0.55	6	11044894	*ELOVL2*	TSS1500	1
cg22736354	0.76	2.9e-15	0.58	6	18122719	*NHLRC1*	1stExon	1
cg06493994	0.62	3.1e-09	0.39	6	25652602	*SCGN*	1stExon	1
cg03236802	0.59	3.0e-08	0.35	6	32055316	*TNXB*	Body	1
cg18651026	-0.59	2.5e-08	0.35	6	33140660	*COL11A2*	Body	1
cg08541518	0.68	1.9e-11	0.47	6	69942892	*BAI3*	Body	1
cg01850269	0.59	2.7e-08	0.35	6	100903227	*SIM1*	Body	1
cg03350900	0.65	5.1e-10	0.42	6	107955689	*SOBP*	Body	1
cg02872426	-0.65	4.8e-10	0.42	6	110736772	*DDO*	TSS200	1
cg06413398	-0.57	8.5e-08	0.33	6	110736865	*DDO*	TSS200	1
cg07164639	-0.59	4.0e-08	0.34	6	110736958	*DDO*	TSS1500	1
cg14956327	-0.58	4.7e-08	0.34	6	110737053	*DDO*	TSS1500	1
cg20979799	0.58	6.3e-08	0.34	6	117198560	*RFX6*	1stExon	1
cg12597389	0.57	1.0e-07	0.33	7	8482235	*NXPH1*	Body	1
cg25962015	-0.58	8.4e-08	0.33	7	26579291	*KIAA0087*	TSS1500	0

cg24853724	0.62	3.6e-09	0.39	7	28997403	*TRIL*	1stExon	1
cg09124496	-0.59	3.6e-08	0.35	7	41735851	*LOC285954*	Body	1
cg10092878	0.59	3.6e-08	0.35	7	73038859	*MLXIPL*	1stExon	0
cg00503840	0.56	2.2e-07	0.31	7	96650509	*DLX5*	Body	1
cg01287975	0.58	5.4e-08	0.34	7	97361241	*TAC1*	TSS200	1
cg08904363	0.61	1.1e-08	0.37	7	102790119	*NAPEPLD*	TSS1500	0
cg04528819	0.56	2.0e-07	0.31	7	130418315	*KLF14*	1stExon	1
cg00094518	0.63	1.7e-09	0.4	7	130418549	*KLF14*	1stExon	0
cg14361627	0.75	1.6e-14	0.56	7	130419116	*KLF14*	TSS1500	1
cg07955995	0.6	1.4e-08	0.36	7	130419159	*KLF14*	TSS1500	1
cg00292135	0.63	1.5e-09	0.4	7	156433068	*C7orf13*	Body	0
cg07927379	0.63	1.6e-09	0.4	7	156433108	*C7orf13*	Body	1
cg23636833	-0.57	1.2e-07	0.32	7	157514822	*PTPRN2*	Body	0
cg26330518	0.58	6.2e-08	0.34	8	24770342	*NEFM*	TSS1500	0
cg07502389	0.66	1.5e-10	0.44	8	24771259	*NEFM*	TSS200	1
cg18267374	0.61	1.0e-08	0.37	8	24771273	*NEFM*	TSS1500	1
cg07583137	-0.56	2.1e-07	0.31	8	82644012	*CHMP4C*	TSS1500	1
cg26290632	0.65	4.2e-10	0.42	8	91094847	*CALB1*	1stExon	1
cg04436528	-0.61	5.6e-09	0.38	8	143554875	*BAI1*	Body	0
cg21159778	0.59	2.7e-08	0.35	9	117266918	*DFNB31*	TSS1500	1
cg14058848	-0.59	4.2e-08	0.34	9	130479114	*TTC16*	Body	0
cg09642020	-0.61	1.1e-08	0.37	9	139379173	*C9orf163*	1stExon	1
cg12610471	0.59	4.1e-08	0.34	10	22634199	*SPAG6*	TSS200	1

cg12377139	0.6	1.5e-08	0.36	10	22634218	*SPAG6*	TSS200	1
cg16541931	0.59	3.8e-08	0.34	10	25463757	*LOC100128811*	Body	1
cg04599297	0.58	6.0e-08	0.34	10	26505442	*GAD2*	5'UTR	0
cg24350475	-0.58	8.1e-08	0.33	10	30316933	*KIAA1462*	Body	1
cg22796704	-0.65	2.6e-10	0.43	10	49673534	*ARHGAP22*	Body	1
cg10056132	0.67	4.3e-11	0.46	10	50887731	*C10orf53*	5'UTR	1
cg18738190	-0.57	1.2e-07	0.32	10	73740291	*CHST3*	5'UTR	1
cg14209784	-0.62	4.8e-09	0.38	10	88729861	*AGAP11*	TSS1500	1
cg19991948	-0.6	1.5e-08	0.36	10	121335173	*TIAL1*	3'UTR	1
cg20012537	-0.6	1.3e-08	0.36	10	134928794	*GPR123*	Body	1
cg07080372	-0.71	1.7e-12	0.5	11	796607	*SLC25A22*	TSS1500	1
cg05267118	-0.62	5.2e-09	0.38	11	2907884	*CDKN1C*	TSS1500	0
cg23091758	0.59	2.9e-08	0.35	11	9025767	*NRIP3*	TSS200	1
cg05923914	-0.56	2.3e-07	0.31	11	45792610	*DKFZp779M0652*	TSS1500	1
cg19589811	0.62	5.4e-09	0.38	11	82443961	*FAM181B*	1stExon	0
cg00202702	0.57	8.5e-08	0.33	11	101918157	*C11orf70*	TSS200	1
cg02423574	-0.57	9.4e-08	0.33	11	119066560	*CCDC153*	1stExon	1
cg11141380	0.56	2.2e-07	0.31	11	122852417	*BSX*	TSS200	0
cg16983588	-0.61	9.8e-09	0.37	11	129793108	*PRDM10*	Body	1
cg08622677	0.64	9.4e-10	0.41	12	3601306	*PRMT8*	Body	1
cg19056004	-0.63	1.8e-09	0.4	12	7023262	*LRRC23*	3'UTR	1
cg04604946	-0.61	8.0e-09	0.37	12	7023352	*LRRC23*	3'UTR	1
cg15626285	-0.62	3.3e-09	0.39	12	7167781	*C1S*	TSS200	1

cg13942826	-0.57	1.4e-07	0.32	12	11002609	*PRR4*	TSS1500	1
cg05242244	-0.62	5.5e-09	0.38	12	53553086	*CSAD*	Body	1
cg17436656	-0.6	2.1e-08	0.36	12	53627106	*RARG*	TSS1500	1
cg22358580	0.58	8.1e-08	0.33	12	54426725	*HOXC4*	5'UTR	1
cg18473521	0.6	1.4e-08	0.36	12	54448265	*HOXC4*	Body	1
cg25778262	0.62	5.5e-09	0.38	12	69327449	*CPM*	TSS1500	0
cg11649376	-0.73	1.1e-13	0.54	12	81473234	*ACSS3*	Body	1
cg08888956	-0.65	3.5e-10	0.42	12	86267839	*NTS*	TSS1500	0
cg12878812	0.64	6.9e-10	0.41	12	119419696	*SRRM4*	1stExon	1
cg11826475	-0.58	6.7e-08	0.33	12	120704006	*PXN*	TSS1500	1
cg09972864	0.67	5.4e-11	0.45	13	22248760	*FGF9*	Body	0
cg18236477	0.6	1.2e-08	0.37	13	26043066	*ATP8A2*	Body	1
cg26685941	-0.58	6.3e-08	0.34	13	95952902	*ABCC4*	Body	1
cg05412028	-0.61	7.6e-09	0.37	13	95952937	*ABCC4*	Body	1
cg00743094	0.56	2.1e-07	0.31	13	100547968	*CLYBL*	3'UTR	0
cg07416237	0.57	1.4e-07	0.32	13	100548078	*CLYBL*	3'UTR	1
cg13917662	0.56	2.1e-07	0.31	13	100548513	*CLYBL*	3'UTR	1
cg05404236	0.56	2.4e-07	0.31	13	110437093	*IRS2*	1stExon	1
cg25078444	0.59	3.0e-08	0.35	14	29235193	*FOXG1*	TSS1500	1
cg01302656	0.64	7.8e-10	0.41	14	29254853	*C14orf23*	Body	1
cg07850604	0.65	2.7e-10	0.43	14	36003443	*INSM2*	5'UTR	1
cg14836018	-0.61	6.8e-09	0.37	14	51706425	*TMX1*	TSS1500	1
cg02583151	0.57	1.0e-07	0.33	14	61116211	*SIX1*	TSS200	0

cg06934523	-0.61	5.7e-09	0.38	14	70077930	*KIAA0247*	TSS1500	0
cg23998119	0.61	8.4e-09	0.37	14	77600217	*ZDHHC22*	Body	1
cg15480367	0.6	2.2e-08	0.35	14	93389485	*CHGA*	5'UTR	1
cg12422450	0.57	1.4e-07	0.32	14	93389891	*CHGA*	Body	1
cg15341124	0.59	2.4e-08	0.35	14	102027734	*DIO3*	5'UTR	1
cg01763090	0.71	1.9e-12	0.5	15	31775406	*OTUD7A*	3'UTR	1
cg04875128	0.78	2.6e-16	0.61	15	31775895	*OTUD7A*	Body	1
cg21296230	0.57	1.6e-07	0.32	15	33010536	*GREM1*	5'UTR	1
cg25826226	0.56	2.2e-07	0.31	15	41953061	*MGA*	5'UTR	0
cg16717122	0.57	1.0e-07	0.33	15	51973920	*SCG3*	5'UTR	1
cg07931844	-0.6	1.5e-08	0.36	15	72102213	*NR2E3*	TSS1500	1
cg21801378	0.61	6.3e-09	0.38	15	72612125	*BRUNOL6*	1stExon	1
cg20809087	0.56	2.0e-07	0.32	15	72612221	*BRUNOL6*	5'UTR	1
cg27552679	0.6	1.3e-08	0.36	15	74425757	*ISLR2*	Body	0
cg27569300	0.66	1.3e-10	0.44	15	99645065	*SYNM*	TSS1500	1
cg01797043	-0.61	6.3e-09	0.38	16	2004686	*RPL3L*	TSS200	1
cg11220950	0.57	1.0e-07	0.33	16	2042693	*SYNGR3*	Body	1
cg27269561	-0.56	1.9e-07	0.32	16	3072713	*HCFC1R1*	3'UTR	0
cg03136323	-0.56	2.2e-07	0.31	16	19195100	*SYT17*	Body	0
cg00448560	-0.61	6.0e-09	0.38	16	49525727	*ZNF423*	Body	1
cg04229059	-0.6	2.2e-08	0.35	16	58718971	*SLC38A7*	TSS1500	1
cg06279276	0.61	6.2e-09	0.38	16	67184164	*B3GNT9*	Body	1
cg09636661	-0.58	6.2e-08	0.34	16	69743901	*NQO1*	3'UTR	1

cg06086177	-0.58	4.8e-08	0.34	16	73090646	*ZFHX3*	5'UTR	0
cg04987608	0.59	3.0e-08	0.35	16	75300043	*BCAR1*	TSS200	0
cg00551679	0.57	1.3e-07	0.32	16	86547530	*FOXF1*	3'UTR	0
cg04503319	-0.58	5.8e-08	0.34	16	89368367	*ANKRD11*	Body	1
cg17457912	-0.57	1.4e-07	0.32	17	1617102	*C17orf91*	Body	1
cg10137837	0.62	3.9e-09	0.38	17	6926742	*BCL6B*	5'UTR	1
cg13029847	0.6	2.2e-08	0.35	17	27333273	*SEZ6*	TSS200	1
cg14692377	0.69	6.5e-12	0.48	17	28562685	*SLC6A4*	1stExon	1
cg06247837	-0.57	1.0e-07	0.33	17	37820135	*TCAP*	TSS1500	1
cg22156456	-0.58	6.5e-08	0.34	17	39844239	*EIF1*	TSS1500	1
cg24212517	-0.57	1.4e-07	0.32	17	42428168	*GRN*	Body	0
cg12892303	0.56	1.8e-07	0.32	17	42733592	*C17orf104*	TSS200	1
cg12781700	0.57	8.7e-08	0.33	17	42733729	*C17orf104*	TSS200	0
cg20396069	-0.58	7.4e-08	0.33	17	46908122	*CALCOCO2*	TSS1500	0
cg24847230	-0.63	1.7e-09	0.4	17	46986807	*UBE2Z*	Body	1
cg18618815	-0.58	8.4e-08	0.33	17	48275324	*COL1A1*	Body	1
cg11071401	0.73	2.7e-13	0.53	17	48637194	*CACNA1G*	TSS1500	1
cg16477091	0.64	1.1e-09	0.4	17	56833000	*PPM1E*	TSS1500	1
cg07584066	0.62	5.0e-09	0.38	17	57642749	*DHX40*	TSS200	1
cg16969368	0.59	3.0e-08	0.35	17	57642752	*DHX40*	TSS200	0
cg26897150	0.6	2.2e-08	0.35	17	57642763	*DHX40*	TSS200	0
cg07365960	0.57	9.8e-08	0.33	17	72848535	*GRIN2C*	Body	1
cg00439658	0.62	5.3e-09	0.38	17	72848669	*GRIN2C*	Body	1

cg05776388	0.62	4.4e-09	0.38	17	72983414	*CDR2L*	TSS1500	0
cg17024199	-0.58	7.0e-08	0.33	17	74881747	*MGAT5B*	Body	1
cg01502244	-0.57	1.3e-07	0.32	17	78188904	*SGSH*	Body	0
cg19761273	-0.61	7.1e-09	0.37	17	80232096	*CSNK1D*	TSS1500	1
cg23718736	-0.57	1.1e-07	0.33	18	6413908	*L3MBTL4*	5'UTR	1
cg03614193	0.6	1.3e-08	0.36	18	55021542	*ST8SIA3*	Body	1
cg19283806	-0.71	1.4e-12	0.5	18	66389420	*CCDC102B*	5'UTR	1
cg17471102	-0.59	3.6e-08	0.35	19	5851255	*FUT3*	5'UTR	1
cg08694544	0.62	4.3e-09	0.38	19	12946228	*RTBDN*	1stExon	0
cg14556683	0.69	7.3e-12	0.48	19	15342982	*EPHX3*	1stExon	1
cg19784428	-0.6	2.0e-08	0.36	19	16830746	*NWD1*	TSS200	1
cg19344626	-0.58	6.9e-08	0.33	19	16830749	*NWD1*	TSS200	1
cg01580888	0.57	1.5e-07	0.32	19	33556060	*RHPN2*	TSS1500	1
cg05346491	-0.6	1.9e-08	0.36	19	48917104	*GRIN2D*	Body	1
cg04731544	0.57	1.1e-07	0.33	19	49004834	*LMTK3*	Body	1
cg19593767	0.58	5.8e-08	0.34	20	42544666	*TOX2*	TSS200	1
cg19702785	0.71	1.9e-12	0.5	20	43727089	*KCNS1*	Body	1
cg06369624	0.57	9.6e-08	0.33	20	43727355	*KCNS1*	Body	0
cg07547549	0.65	4.7e-10	0.42	20	44658225	*SLC12A5*	Body	1
cg00387658	-0.62	5.3e-09	0.38	20	54986793	*CASS4*	TSS1500	1
cg14374829	0.58	4.4e-08	0.34	21	33244742	*HUNK*	TSS1500	0
cg07127410	-0.58	7.7e-08	0.33	22	29427851	*ZNRF3*	Body	0
cg21064451	-0.57	1.2e-07	0.32	22	38071534	*LGALS1*	TSS200	0

							*Beta: standardized regression coefficients *: knwon age-related CpG (No = 0; Yes =1)*	

Supplementary Table S2 Age-related eCpGs

CpG	Transcript	CpG		Transcript		CpG x Transcript		R2	CHR	MAPINFO	Gene	feature	r[a]	index	Known[b]
		Beta	P	Beta	P	Beta	P								
cg07198402	ILMN_1749667	8.69	2.4e-02	10.48	2.0e-02	-14.3	2.0e-02	0.17	1	228395145	*OBSCN*	TSS1500	0.07	y	0
cg07600278	ILMN_1749667	8.39	4.6e-02	14.71	4.4e-02	-16.67	4.4e-02	0.06	1	228548163	*OBSCN*	Body	-0.04	n	0
cg13539203	ILMN_1768483	9.55	7.8e-03	10.02	7.8e-03	-14.08	7.1e-03	0.12	2	26950545	*KCNK3*	Body	0.01	y	0
cg10666081	ILMN_2355831	-6.92	8.4e-03	-12.79	1.2e-02	14.17	1.1e-02	0.17	2	105985002	*FHL2*	Body	-0.05	y	0
cg06907053	ILMN_1668411	6.63	3.2e-02	2.37	2.9e-02	-6.96	3.5e-02	0.09	2	106015869	*FHL2*	TSS200	0.04	n	0
cg08239282	ILMN_2355831	-7.99	1.5e-02	-19.35	1.8e-02	19.98	1.7e-02	0.12	2	106026162	*FHL2*	5'UTR	-0.13	n	0
cg08239282	ILMN_1668411	-7.22	2.8e-02	-17.36	3.4e-02	18.88	3.3e-02	0.11	2	106026162	*FHL2*	5'UTR	0.01	n	0
cg12336777	ILMN_1688698	4.85	2.8e-02	1.52	1.5e-02	-5.22	2.9e-02	0.1	2	145210222	*ZEB2*	Body	0.18	n	0
cg12571570	ILMN_1820767	-8.91	2.8e-02	-2.76	4.9e-02	9	3.0e-02	0.16	2	145267984	*ZEB2*	Body	-0.09	n	0
cg23095192	ILMN_1820767	-8.89	1.9e-02	-3.94	3.2e-02	8.89	2.2e-02	0.2	2	145271307	*ZEB2*	Body	-0.19	y	0
cg07246225	ILMN_1688698	5.49	2.6e-02	1.77	1.8e-02	-5.53	3.2e-02	0.14	2	145277659	*ZEB2*	Body	0.01	n	0
cg14421309	ILMN_1820767	-10.41	4.5e-03	-1.76	1.4e-02	10.87	4.2e-03	0.19	2	145277734	*ZEB2*	Body	0.1	n	0
cg12974733	ILMN_1739366	-5.3	1.2e-02	-1.24	1.5e-02	5.79	1.0e-02	0.1	2	162273524	*TBR1*	1stExon	0.19	n	0
cg22743761	ILMN_1739366	-5.7	3.2e-02	-2.22	2.7e-02	6.85	2.6e-02	0.11	2	162273648	*TBR1*	Body	0.26	y	0
cg03578473	ILMN_1669425	-9.72	1.3e-02	-1.15	1.3e-02	9.9	1.2e-02	0.09	2	182546504	*NEUROD1*	TSS1500	0.05	y	0
cg20671534	ILMN_1658576	-6.04	6.5e-03	-2.56	1.2e-02	6.65	8.8e-03	0.15	2	220174629	*PTPRN*	TSS1500	0.12	y	0
cg21126969	ILMN_2391750	4.56	4.5e-02	7.6	2.9e-02	-10.02	3.1e-02	0.17	3	53030684	*SFMBT1*	5'UTR	0.28	n	0
cg12962167	ILMN_2391750	5.11	3.2e-02	13.97	3.0e-02	-14.23	3.1e-02	0.07	3	53033115	*SFMBT1*	5'UTR	-0.13	n	0
cg17761990	ILMN_1741585	8.07	6.4e-03	11.07	5.7e-03	-14.09	5.4e-03	0.14	3	53042940	*SFMBT1*	5'UTR	0.03	n	0
cg17761990	ILMN_2391750	8.32	1.4e-03	14.69	1.0e-03	-17.13	1.1e-03	0.17	3	53042940	*SFMBT1*	5'UTR	0.03	y	0
cg24445507	ILMN_2391750	4.55	2.6e-02	11.38	1.9e-02	-12.15	1.9e-02	0.12	3	53044901	*SFMBT1*	5'UTR	-0.04	n	0
cg04503182	ILMN_2391750	4.36	3.5e-02	8.54	2.6e-02	-9.05	2.5e-02	0.13	3	53078218	*SFMBT1*	5'UTR	-0.16	n	0
cg20332195	ILMN_1682449	-10.72	9.2e-03	-2.15	9.0e-03	11.19	7.3e-03	0.2	4	10459929	*ZNF518B*	TSS1500	-0.04	y	0

cg03463279	ILMN_1774948	6.11	4.4e-02	2.1	2.7e-02	-6.44	4.4e-02	0.1	4	15004942	*CPEB2*	TSS1500	0.04	n	0
cg19929852	ILMN_1774948	9.08	1.0e-02	13.15	8.6e-03	-15.21	9.6e-03	0.14	4	15068643	*CPEB2*	3'UTR	-0.08	y	0
cg10512498	ILMN_1746552	6.42	2.4e-02	7.42	2.2e-02	-9.27	2.1e-02	0.1	4	176986333	*WDR17*	TSS1500	-0.14	n	0
cg18443378	ILMN_1746552	-5.65	4.2e-02	-1.52	3.1e-02	6.09	3.2e-02	0.16	4	176986950	*WDR17*	TSS200	-0.04	y	0
cg14782559	ILMN_1721178	-10.87	2.5e-03	-0.79	8.2e-03	10.9	2.9e-03	0.14	6	33131893	*COL11A2*	Body	0.21	n	0
cg20506659	ILMN_1721178	8.82	4.9e-02	4.65	4.4e-02	-9.37	4.1e-02	0.16	6	33132943	*COL11A2*	Body	-0.22	n	0
cg02667414	ILMN_1748166	9.52	3.5e-02	11.59	3.5e-02	-14.54	3.3e-02	0.09	6	33133517	*COL11A2*	Body	-0.08	n	0
cg06924878	ILMN_1748166	8.56	1.9e-02	3.81	2.0e-02	-9.43	1.7e-02	0.12	6	33134045	*COL11A2*	Body	-0.04	n	0
cg14812123	ILMN_1748166	6.99	3.3e-02	12.68	3.2e-02	-14.46	3.0e-02	0.09	6	33135284	*COL11A2*	Body	-0.03	n	0
cg00933415	ILMN_1748166	7.66	1.9e-02	9.42	1.7e-02	-12.81	1.6e-02	0.14	6	33135453	*COL11A2*	Body	0.07	n	0
cg25482983	ILMN_1748166	7.71	4.6e-02	7.81	4.4e-02	-10.68	4.1e-02	0.1	6	33135756	*COL11A2*	Body	-0.09	n	0
cg22748722	ILMN_1748166	7.31	4.1e-02	8.9	3.8e-02	-11.59	3.6e-02	0.11	6	33135868	*COL11A2*	Body	-0.03	n	0
cg24158878	ILMN_1721178	10.62	2.7e-02	2.2	2.9e-02	-10.58	2.9e-02	0.07	6	33137570	*COL11A2*	Body	-0.08	n	0
cg02673107	ILMN_1721178	14.88	3.3e-04	5.68	3.2e-04	-15.09	2.9e-04	0.19	6	33138131	*COL11A2*	Body	-0.18	n	0
cg18651026	ILMN_1748166	5.35	4.3e-02	3.16	2.9e-02	-6.87	2.5e-02	0.4	6	33140660	*COL11A2*	Body	0.04	y	1
cg20974826	ILMN_1721178	8.78	3.0e-02	5.44	2.6e-02	-9.7	2.5e-02	0.15	6	33141040	*COL11A2*	Body	-0.18	n	1
cg00350615	ILMN_1721178	8.5	5.0e-02	3.64	4.7e-02	-8.98	4.5e-02	0.09	6	33141152	*COL11A2*	Body	-0.13	n	1
cg13224161	ILMN_1721178	12.94	6.2e-03	2.39	6.6e-03	-13.01	6.0e-03	0.1	6	33141279	*COL11A2*	Body	-0.09	n	0
cg00115458	ILMN_1721178	11.18	5.6e-03	2.35	5.5e-03	-11.47	4.9e-03	0.14	6	33141305	*COL11A2*	Body	-0.06	n	0
cg11960243	ILMN_1721178	12.85	2.8e-03	1.34	3.6e-03	-12.59	3.1e-03	0.13	6	33143135	*COL11A2*	Body	-0.16	n	0
cg21771125	ILMN_1721178	-8.02	3.5e-02	-8	4.4e-02	11.41	4.4e-02	0.17	6	33149047	*COL11A2*	Body	0.06	n	0
cg03260211	ILMN_1721178	-8.1	3.9e-02	-7.37	4.5e-02	12.22	4.3e-02	0.09	6	33151921	*COL11A2*	Body	0.27	n	0
cg04182805	ILMN_1748166	9.23	3.0e-02	19.44	2.7e-02	-22.09	2.7e-02	0.11	6	33154830	*COL11A2*	Body	0.05	n	0
cg21509966	ILMN_1748166	5.94	2.8e-02	10.88	2.6e-02	-12.61	2.5e-02	0.1	6	33156463	*COL11A2*	Body	0.01	n	0
cg11835806	ILMN_1721178	-8.41	2.9e-02	-12.32	3.3e-02	15.44	3.3e-02	0.1	6	33156836	*COL11A2*	Body	0.09	n	0
cg24936204	ILMN_1721178	7.8	4.1e-02	5.24	3.7e-02	-8.62	3.4e-02	0.14	6	33157138	*COL11A2*	Body	-0.23	n	0
cg16507569	ILMN_1721178	-8.56	1.3e-02	-0.86	3.5e-02	8.57	1.7e-02	0.15	6	33158020	*COL11A2*	Body	0.27	n	0
cg19628686	ILMN_1721178	-11.06	1.2e-02	-9.5	1.4e-02	15.26	1.4e-02	0.12	6	33158503	*COL11A2*	Body	0.12	n	0

cg11700230	ILMN_1721178	6.58	3.6e-02	3.51	3.9e-02	-6.63	4.0e-02	0.07	6	33159353	*COL11A2*	Body	-0.22	n	0
cg26385062	ILMN_1721178	6.61	2.6e-02	3.44	2.7e-02	-6.78	2.7e-02	0.07	6	33159361	*COL11A2*	Body	-0.2	n	0
cg02452416	ILMN_1748166	-7.48	4.9e-02	-1.09	3.2e-02	7.66	4.5e-02	0.09	6	33160450	*COL11A2*	TSS1500	-0.04	n	0
cg05615552	ILMN_1748166	-7.62	3.3e-02	-0.95	2.2e-02	7.67	3.0e-02	0.09	6	33160952	*COL11A2*	TSS1500	-0.15	n	0
cg02764478	ILMN_1696279	6.66	3.7e-02	1.85	4.9e-02	-6.46	4.5e-02	0.14	6	100904316	*SIM1*	Body	-0.1	y	1
cg03511031	ILMN_1728844	-6.26	3.2e-02	-9.48	3.3e-02	10.72	3.2e-02	0.07	7	157460477	*PTPRN2*	Body	-0.13	n	0
cg22623223	ILMN_1728844	7.14	2.0e-02	1.77	2.0e-02	-7.25	2.3e-02	0.1	7	157484045	*PTPRN2*	Body	0.04	n	0
cg11872672	ILMN_1728844	-7.36	2.6e-02	-5.7	3.7e-02	7.75	3.9e-02	0.36	7	157514730	*PTPRN2*	Body	-0.23	y	0
cg04237822	ILMN_1728844	-5.79	3.6e-02	-3.63	3.7e-02	6.74	3.3e-02	0.07	7	157523541	*PTPRN2*	Body	-0.06	n	0
cg25821143	ILMN_1728844	-6.03	2.2e-02	-6.93	2.5e-02	8.97	2.4e-02	0.08	7	157529705	*PTPRN2*	Body	-0.04	n	0
cg24935409	ILMN_1728844	7.27	3.0e-02	2.32	2.7e-02	-7.55	3.0e-02	0.07	7	157667762	*PTPRN2*	Body	-0.02	n	0
cg09845489	ILMN_1728844	7.95	4.8e-03	1.46	5.1e-03	-8.18	5.3e-03	0.12	7	157667853	*PTPRN2*	Body	0.14	n	0
cg09350411	ILMN_1728844	7.84	9.6e-03	4	9.1e-03	-8.99	9.9e-03	0.1	7	157667935	*PTPRN2*	Body	0.07	n	0
cg19013611	ILMN_1728844	8.87	2.4e-03	1.84	2.1e-03	-9.17	2.4e-03	0.13	7	157668051	*PTPRN2*	Body	0.08	n	0
cg15298059	ILMN_1728844	6.4	1.6e-02	1.25	1.6e-02	-6.54	1.7e-02	0.09	7	157668141	*PTPRN2*	Body	0.1	n	0
cg24241530	ILMN_1728844	-5.44	4.6e-02	-7.93	4.9e-02	9.31	4.7e-02	0.06	7	158061820	*PTPRN2*	Body	-0.07	n	0
cg16419421	ILMN_1728844	-8.45	7.4e-03	-10.72	7.4e-03	12.56	7.0e-03	0.11	7	158073079	*PTPRN2*	Body	-0.17	n	0
cg13409070	ILMN_1728844	6.47	4.4e-02	7.83	3.9e-02	-11.1	4.1e-02	0.07	7	158114558	*PTPRN2*	Body	0.19	n	0
cg18566911	ILMN_1728844	-6.71	3.2e-02	-14.74	3.6e-02	14.69	3.6e-02	0.08	7	158183784	*PTPRN2*	Body	-0.24	n	0
cg20764780	ILMN_1728844	-7.78	3.4e-02	-3.18	3.6e-02	8.35	3.2e-02	0.08	7	158187725	*PTPRN2*	Body	-0.06	n	0
cg15035217	ILMN_1728844	-7.24	4.0e-02	-10.89	4.6e-02	13.04	4.5e-02	0.09	7	158235051	*PTPRN2*	Body	0	n	0
cg02512724	ILMN_1728844	10.15	6.5e-03	9.86	6.5e-03	-12.73	6.8e-03	0.11	7	158343573	*PTPRN2*	Body	-0.18	n	0
cg00841760	ILMN_1728844	-8.54	2.5e-02	-2.71	2.8e-02	8.74	2.4e-02	0.07	7	158362966	*PTPRN2*	Body	-0.09	n	0
cg26180501	ILMN_1728844	-7.56	3.5e-02	-3.93	4.2e-02	8.11	3.9e-02	0.08	7	158363265	*PTPRN2*	Body	-0.09	n	0
cg21325485	ILMN_1728844	-7.2	2.7e-02	-3.91	3.2e-02	8.12	2.9e-02	0.09	7	158364932	*PTPRN2*	Body	0	n	0
cg17949162	ILMN_1796855	13.11	1.6e-02	1.51	9.1e-03	-13.17	1.7e-02	0.11	10	121355153	*TIAL1*	Body	0.03	y	0
cg19262958	ILMN_1687958	4.49	4.8e-02	8.21	4.2e-02	-9.57	3.8e-02	0.14	11	792861	*SLC25A22*	Body	-0.02	y	0
cg10970251	ILMN_1687958	-7.57	2.6e-02	-2.73	1.7e-02	7.66	2.6e-02	0.11	11	796421	*SLC25A22*	TSS1500	-0.11	n	0

cg11718501	ILMN_1666310	5.18	4.2e-02	1.13	2.3e-02	-5.09	4.1e-02	0.08	11	122850972	*BSX*	Body	-0.21	y	0
cg22345428	ILMN_2352295	-7	4.7e-02	-1.32	3.7e-02	7.23	4.2e-02	0.1	11	129781496	*PRDM10*	Body	-0.06	n	0
cg24937685	ILMN_2352295	9.73	2.0e-02	9.39	2.1e-02	-13.3	2.0e-02	0.08	11	129806002	*PRDM10*	Body	-0.04	n	0
cg12284171	ILMN_2352295	9.04	4.0e-02	11.04	3.9e-02	-14.15	3.8e-02	0.08	11	129814770	*PRDM10*	Body	-0.03	n	0
cg14082919	ILMN_2352295	11.16	8.2e-03	14.14	7.3e-03	-16.69	6.8e-03	0.17	11	129814820	*PRDM10*	Body	-0.17	y	0
cg12409074	ILMN_2352295	-11.66	1.4e-02	-3.15	1.3e-02	11.44	1.5e-02	0.1	11	129870648	*PRDM10*	5'UTR	-0.16	n	0
cg03198733	ILMN_1781626	-8.1	3.3e-02	-8.59	3.8e-02	11.75	3.8e-02	0.11	12	7167584	*C1S*	TSS1500	0.02	n	0
cg13053396	ILMN_1781626	-10.19	4.5e-03	-8.53	5.1e-03	12.23	5.4e-03	0.15	12	7168545	*C1S*	5'UTR	-0.13	y	0
cg20378628	ILMN_1781626	-8.86	1.5e-02	-11.74	1.8e-02	15.48	1.8e-02	0.14	12	7169154	*C1S*	5'UTR	0.14	n	0
cg22509041	ILMN_2119692	5.42	4.1e-02	1.18	2.4e-02	-5.6	4.3e-02	0.09	12	53574524	*CSAD*	TSS200	0.15	y	0
cg15817960	ILMN_1719975	-4.13	1.5e-02	-1.1	2.2e-02	4.58	1.5e-02	0.08	12	54409599	*HOXC4*	TSS1500	0.31	n	0
cg20381985	ILMN_1719975	-4.94	2.5e-02	-3.59	3.3e-02	5.73	3.3e-02	0.12	12	54410662	*HOXC4*	5'UTR	-0.06	n	0
cg09481972	ILMN_1719975	-5.66	1.5e-02	-2.89	1.4e-02	6.25	1.3e-02	0.1	12	54410666	*HOXC4*	5'UTR	-0.08	n	0
cg10918927	ILMN_1719975	-5	8.1e-03	-3.62	7.7e-03	5.95	7.2e-03	0.1	12	54411193	*HOXC4*	5'UTR	-0.1	n	0
cg16765387	ILMN_1719975	-5.3	7.1e-04	-3.14	8.3e-04	5.9	7.2e-04	0.15	12	54411245	*HOXC4*	5'UTR	-0.09	y	0
cg00506343	ILMN_1719975	-3.64	3.7e-02	-1.64	3.0e-02	4.29	2.8e-02	0.11	12	54421033	*HOXC4*	5'UTR	0.07	n	0
cg19182597	ILMN_1719975	-3.71	1.9e-02	-1.35	2.0e-02	4.17	1.7e-02	0.08	12	54426670	*HOXC4*	5'UTR	0.14	n	0
cg15244786	ILMN_1719975	3.47	4.8e-02	2.16	4.6e-02	-4.36	4.6e-02	0.06	12	54446279	*HOXC4*	5'UTR	0.15	n	0
cg22370252	ILMN_1719975	3.57	3.9e-02	1.94	3.7e-02	-4.37	3.7e-02	0.06	12	54446289	*HOXC4*	5'UTR	0.18	n	0
cg14233050	ILMN_1684440	4.64	1.5e-02	15.22	1.3e-02	-15.37	1.4e-02	0.11	12	120651871	*PXN*	Body	-0.09	n	0
cg01866518	ILMN_1684440	4.36	3.2e-02	9.76	2.7e-02	-10.63	2.9e-02	0.09	12	120673235	*PXN*	5'UTR	0.01	n	0
cg04256697	ILMN_1684440	5.15	1.3e-02	3.05	8.5e-03	-6.22	1.2e-02	0.11	12	120688557	*PXN*	Body	0.1	y	0
cg17370322	ILMN_1788457	7.85	1.4e-02	0.88	7.8e-03	-7.95	1.5e-02	0.1	13	95953482	*ABCC4*	Body	0.16	y	0
cg22853485	ILMN_1663538	-4.58	2.1e-02	-1.76	1.5e-02	4.88	2.1e-02	0.09	13	100258524	*CLYBL*	TSS1500	0	n	0
cg01726399	ILMN_1663538	-5.11	2.6e-02	-2.1	1.8e-02	5.56	2.4e-02	0.09	13	100258641	*CLYBL*	TSS1500	0	n	0
cg26867370	ILMN_1663538	4.27	4.4e-02	6.12	4.4e-02	-7.64	4.0e-02	0.08	13	100262973	*CLYBL*	Body	0.01	n	0
cg03261783	ILMN_1663538	4.66	2.3e-02	10.68	2.1e-02	-11.42	1.9e-02	0.1	13	100497453	*CLYBL*	Body	-0.09	n	0
cg26862247	ILMN_1663538	4.36	4.1e-02	14.09	3.6e-02	-15.19	3.5e-02	0.09	13	100497665	*CLYBL*	Body	0.07	n	0

cg00130181	ILMN_1663538	5.02	1.3e-02	9.05	1.2e-02	-10.46	1.1e-02	0.12	13	100517033	*CLYBL*	Body	-0.01	n	0
cg04259706	ILMN_1712088	-7.93	3.8e-02	-9.68	3.8e-02	12.42	3.9e-02	0.06	13	100545517	*CLYBL*	3'UTR	0	n	0
cg00743094	ILMN_1663538	-3.09	4.3e-02	-1.21	7.3e-03	3.9	1.7e-02	0.39	13	100547968	*CLYBL*	3'UTR	0.09	y	0
cg10912240	ILMN_2149946	-5.43	3.2e-02	-1.76	2.4e-02	5.99	2.6e-02	0.11	14	29235907	*FOXG1*	TSS1500	0.06	y	1
cg08198377	ILMN_1691181	-3.6	2.7e-02	-1.84	3.2e-02	3.62	2.8e-02	0.07	14	51707975	*TMX1*	Body	-0.24	y	0
cg06799422	ILMN_2372379	7.08	1.7e-02	1.54	1.7e-02	-7	2.3e-02	0.19	15	41952235	*MGA*	TSS1500	0.07	y	1
cg03752425	ILMN_1808587	5.51	4.3e-02	9.85	3.6e-02	-11.77	3.7e-02	0.09	16	72840504	*ZFHX3*	Body	0.1	n	0
cg08858926	ILMN_1808587	-7.5	8.9e-03	-3.74	1.2e-02	7.97	1.0e-02	0.12	16	72918832	*ZFHX3*	Body	-0.09	y	0
cg10415570	ILMN_1808587	-9.11	7.6e-03	-16.32	8.7e-03	17.43	8.5e-03	0.12	16	73053779	*ZFHX3*	5'UTR	-0.15	n	0
cg03891962	ILMN_1690465	-9.26	9.9e-04	-11.7	1.1e-03	16.49	1.1e-03	0.15	16	89336560	*ANKRD11*	Body	0.23	n	0
cg02033182	ILMN_1690465	-6.41	4.2e-02	-7.52	4.0e-02	10.18	4.1e-02	0.06	16	89337334	*ANKRD11*	Body	0.06	n	0
cg03594907	ILMN_1690465	-7.46	1.8e-02	-15.46	2.3e-02	17.83	2.2e-02	0.13	16	89337953	*ANKRD11*	Body	0.12	n	0
cg09474599	ILMN_1690465	-6.09	3.7e-02	-14.32	3.8e-02	15.78	3.8e-02	0.06	16	89338043	*ANKRD11*	Body	0.04	n	0
cg04381043	ILMN_1690465	-10.15	9.6e-04	-22.02	1.1e-03	24.72	1.1e-03	0.17	16	89341467	*ANKRD11*	Body	0.06	n	0
cg06098690	ILMN_1690465	-11.53	2.3e-04	-30.12	2.5e-04	32.88	2.5e-04	0.18	16	89341682	*ANKRD11*	Body	0.06	n	0
cg10256077	ILMN_1690465	-7.66	3.0e-02	-14.61	2.9e-02	17.7	3.0e-02	0.07	16	89341851	*ANKRD11*	Body	0.18	n	0
cg26479658	ILMN_1690465	-10.83	3.7e-03	-19.06	4.4e-03	22.54	4.4e-03	0.15	16	89342677	*ANKRD11*	Body	0.08	n	0
cg09867358	ILMN_1690465	-8.95	8.5e-04	-20.83	1.0e-03	22.3	1.0e-03	0.16	16	89342851	*ANKRD11*	Body	-0.04	n	0
cg08927754	ILMN_1690465	-6.12	3.5e-02	-8.86	3.8e-02	11.28	3.8e-02	0.07	16	89344197	*ANKRD11*	Body	0.12	n	0
cg26747517	ILMN_1690465	-6.86	1.3e-02	-9.92	1.3e-02	12.05	1.4e-02	0.09	16	89344383	*ANKRD11*	Body	0.01	n	0
cg11051669	ILMN_1690465	-7.72	1.0e-02	-17.16	1.2e-02	19.65	1.2e-02	0.11	16	89346436	*ANKRD11*	Body	0.13	n	0
cg00679681	ILMN_1690465	-14.87	5.5e-05	-41.63	6.1e-05	44.5	6.1e-05	0.22	16	89346491	*ANKRD11*	Body	0.03	n	0
cg04949226	ILMN_1690465	-9.23	4.4e-03	-23.53	5.2e-03	25.4	5.2e-03	0.13	16	89348469	*ANKRD11*	Body	0.02	n	0
cg03362018	ILMN_1690465	-7.22	3.0e-02	-15.58	3.3e-02	17.77	3.3e-02	0.08	16	89348956	*ANKRD11*	Body	0.1	n	0
cg09301471	ILMN_1690465	-6.31	4.3e-02	-13.22	4.9e-02	14.94	4.9e-02	0.09	16	89349945	*ANKRD11*	Body	0.07	n	0
cg02500806	ILMN_1690465	-6.35	3.0e-02	-10.43	3.1e-02	12.32	3.1e-02	0.07	16	89349976	*ANKRD11*	Body	0.03	n	0
cg06636372	ILMN_1690465	-10.23	6.0e-04	-15.95	7.3e-04	19.48	7.3e-04	0.18	16	89349979	*ANKRD11*	Body	0.08	n	0
cg08130612	ILMN_1690465	-5.88	1.8e-02	-11.97	2.2e-02	13.52	2.2e-02	0.12	16	89350233	*ANKRD11*	Body	0.06	n	0

cg00199000	ILMN_1690465	-6.7	3.1e-02	-11.34	3.4e-02	13.66	3.4e-02	0.08	16	89350310	*ANKRD11*	Body	0.1	n	0
cg04937029	ILMN_1690465	-5.69	3.8e-02	-11.03	4.4e-02	12.79	4.4e-02	0.08	16	89351359	*ANKRD11*	Body	0.09	n	0
cg16593941	ILMN_1690465	-8.26	2.0e-02	-19.78	2.6e-02	22.1	2.6e-02	0.19	16	89351893	*ANKRD11*	Body	0.11	n	0
cg02134790	ILMN_1690465	-7.5	3.6e-03	-19.32	4.4e-03	21.89	4.4e-03	0.14	16	89352812	*ANKRD11*	Body	0.18	n	0
cg09274451	ILMN_1690465	-7.96	9.7e-03	-13.99	1.2e-02	16.27	1.2e-02	0.14	16	89353600	*ANKRD11*	Body	0.04	n	0
cg07159469	ILMN_1690465	-6.66	1.7e-02	-19.94	2.2e-02	21.8	2.2e-02	0.14	16	89355005	*ANKRD11*	Body	0.14	n	0
cg03317919	ILMN_1690465	-7.57	1.3e-02	-16.8	1.3e-02	19.34	1.4e-02	0.09	16	89355071	*ANKRD11*	Body	0.14	n	0
cg01875529	ILMN_1690465	-8.84	6.4e-03	-18.53	7.0e-03	21.95	7.0e-03	0.11	16	89355462	*ANKRD11*	Body	0.19	n	0
cg09875780	ILMN_1690465	-6.24	3.7e-02	-15.27	4.5e-02	17.2	4.4e-02	0.11	16	89357086	*ANKRD11*	Body	0.14	n	0
cg09776094	ILMN_1690465	-8.56	1.6e-02	-15.39	1.8e-02	19.01	1.7e-02	0.1	16	89357477	*ANKRD11*	Body	0.2	n	0
cg27207530	ILMN_1690465	-9.21	5.8e-03	-17.17	6.9e-03	21	6.8e-03	0.13	16	89357600	*ANKRD11*	Body	0.21	n	0
cg09220891	ILMN_1690465	-8.04	3.5e-02	-9.55	3.7e-02	13.01	3.8e-02	0.07	16	89357741	*ANKRD11*	Body	0.1	n	0
cg02487202	ILMN_1690465	-8.56	4.4e-03	-14.48	5.3e-03	17.83	5.3e-03	0.14	16	89358232	*ANKRD11*	Body	0.16	n	0
cg03025000	ILMN_1690465	-9.68	4.3e-03	-15.69	5.0e-03	20.01	5.0e-03	0.13	16	89358837	*ANKRD11*	Body	0.21	n	0
cg27267258	ILMN_1690465	-6.83	1.6e-02	-15.93	1.8e-02	18.93	1.8e-02	0.08	16	89359175	*ANKRD11*	Body	0.28	n	0
cg07108351	ILMN_1690465	-8.43	1.5e-03	-18.56	1.8e-03	21.66	1.7e-03	0.15	16	89359631	*ANKRD11*	Body	0.18	n	0
cg00332509	ILMN_1690465	-8.49	5.4e-03	-16.05	6.0e-03	19.51	5.9e-03	0.11	16	89363830	*ANKRD11*	Body	0.2	n	0
cg09607566	ILMN_1690465	-6.93	2.6e-02	-10.52	3.1e-02	13.25	3.1e-02	0.11	16	89367323	*ANKRD11*	Body	0.14	n	0
cg26783123	ILMN_1690465	-6.31	3.9e-02	-7.23	4.7e-02	9.85	4.7e-02	0.11	16	89367859	*ANKRD11*	Body	0.09	n	0
cg16699022	ILMN_1690465	-7.06	1.3e-02	-17.17	1.6e-02	19.38	1.6e-02	0.12	16	89371566	*ANKRD11*	Body	0.14	n	0
cg00711584	ILMN_1690465	-7.35	1.2e-02	-24.95	1.4e-02	26.56	1.4e-02	0.11	16	89373295	*ANKRD11*	Body	0.09	n	0
cg07661162	ILMN_1690465	-6.13	1.3e-02	-11.16	1.6e-02	13.84	1.5e-02	0.1	16	89375203	*ANKRD11*	Body	0.23	n	0
cg03615921	ILMN_1690465	-8.33	7.6e-03	-22.05	9.8e-03	24.46	9.7e-03	0.16	16	89375284	*ANKRD11*	Body	0.13	n	0
cg02415470	ILMN_1690465	-6.3	2.4e-02	-10.52	2.4e-02	13.77	2.4e-02	0.07	16	89383614	*ANKRD11*	5'UTR	0.3	n	0
cg26546229	ILMN_1690465	-8.22	4.5e-03	-18.25	5.3e-03	20.17	5.4e-03	0.14	16	89385611	*ANKRD11*	5'UTR	0.03	n	0
cg08888321	ILMN_1690465	-7.26	4.8e-03	-13.39	5.4e-03	15.32	5.4e-03	0.12	16	89385892	*ANKRD11*	5'UTR	0.02	n	0
cg27247807	ILMN_1690465	-9.45	5.6e-04	-16.47	6.1e-04	20.41	6.1e-04	0.16	16	89387225	*ANKRD11*	5'UTR	0.19	n	0
cg27300957	ILMN_1690465	-9.8	7.5e-04	-11.77	1.0e-03	15.85	1.0e-03	0.21	16	89387418	*ANKRD11*	5'UTR	0.1	n	0

cg09420319	ILMN_1690465	-7.83	4.6e-02	-17.1	4.8e-02	19.92	4.8e-02	0.06	16	89387569	*ANKRD11*	5'UTR	0.17	n	0
cg08311935	ILMN_1690465	-7.5	5.2e-03	-9.65	5.8e-03	12.74	5.8e-03	0.12	16	89387805	*ANKRD11*	5'UTR	0.1	n	0
cg05504729	ILMN_1690465	-7.94	1.8e-02	-4.04	2.1e-02	8.9	2.1e-02	0.11	16	89399490	*ANKRD11*	5'UTR	0.05	n	0
cg07054502	ILMN_1690465	-10.71	3.3e-04	-7.8	4.4e-04	13.88	4.3e-04	0.2	16	89405478	*ANKRD11*	5'UTR	0.13	n	0
cg06873916	ILMN_1690465	-7.86	2.7e-02	-17.56	3.1e-02	19.98	3.1e-02	0.11	16	89410196	*ANKRD11*	5'UTR	0.12	n	0
cg05071195	ILMN_1690465	-7.12	7.7e-03	-10.31	7.9e-03	13.55	7.9e-03	0.1	16	89411232	*ANKRD11*	5'UTR	0.19	n	0
cg01271982	ILMN_1690465	-8.03	2.4e-03	-16.39	2.8e-03	18.96	2.8e-03	0.13	16	89417545	*ANKRD11*	5'UTR	0.11	n	0
cg00911267	ILMN_1690465	-10.89	4.1e-04	-10.97	5.4e-04	16.4	5.3e-04	0.2	16	89418486	*ANKRD11*	5'UTR	0.15	n	0
cg05507697	ILMN_1690465	-9.42	7.2e-05	-11.21	9.0e-05	16.09	8.7e-05	0.21	16	89421700	*ANKRD11*	5'UTR	0.22	n	0
cg06411447	ILMN_1690465	-9.02	4.9e-03	-8.64	6.5e-03	13.48	6.2e-03	0.17	16	89421749	*ANKRD11*	5'UTR	0.2	n	0
cg01619533	ILMN_1690465	-8.43	2.8e-03	-11.46	3.1e-03	15.86	3.1e-03	0.13	16	89421770	*ANKRD11*	5'UTR	0.26	n	0
cg02749356	ILMN_1690465	-11.39	1.0e-04	-22.64	1.2e-04	26.85	1.2e-04	0.21	16	89421929	*ANKRD11*	5'UTR	0.16	n	0
cg26533023	ILMN_1690465	-8.69	3.9e-03	-22.59	4.5e-03	25.45	4.5e-03	0.12	16	89422171	*ANKRD11*	5'UTR	0.17	n	0
cg00345343	ILMN_1690465	-6.81	9.4e-03	-13.4	1.4e-02	15.61	1.4e-02	0.2	16	89422185	*ANKRD11*	5'UTR	0.12	n	0
cg27096292	ILMN_1690465	-9.3	1.4e-03	-19.94	1.8e-03	23.07	1.7e-03	0.16	16	89422212	*ANKRD11*	5'UTR	0.14	n	0
cg00948881	ILMN_1690465	-7.3	3.3e-02	-15.04	3.8e-02	17.18	3.8e-02	0.11	16	89433544	*ANKRD11*	5'UTR	0.09	n	0
cg26877336	ILMN_1690465	-5.83	2.0e-02	-11.02	2.2e-02	12.84	2.2e-02	0.08	16	89433760	*ANKRD11*	5'UTR	0.09	n	0
cg06660116	ILMN_1690465	-6.98	1.4e-02	-13.43	1.4e-02	16.38	1.4e-02	0.08	16	89433876	*ANKRD11*	5'UTR	0.21	n	0
cg08420923	ILMN_1690465	-10.18	2.6e-03	-9.58	2.3e-03	15.23	2.4e-03	0.13	16	89436447	*ANKRD11*	5'UTR	0.18	n	1
cg09033641	ILMN_1690465	-7.63	3.5e-02	-10.07	3.8e-02	13.47	3.8e-02	0.08	16	89436838	*ANKRD11*	5'UTR	0.16	n	0
cg10515125	ILMN_1690465	-11.73	1.5e-04	-30.19	1.7e-04	33.38	1.7e-04	0.19	16	89437361	*ANKRD11*	5'UTR	0.1	n	0
cg03633073	ILMN_1690465	-8	1.4e-02	-33.21	1.8e-02	34.58	1.8e-02	0.18	16	89437518	*ANKRD11*	5'UTR	0.07	n	0
cg12069856	ILMN_1690465	-9.24	3.8e-03	-16.98	5.1e-03	20.37	5.1e-03	0.19	16	89438504	*ANKRD11*	5'UTR	0.15	n	0
cg07447015	ILMN_1690465	-9.24	2.4e-03	-20.19	2.6e-03	23.64	2.6e-03	0.13	16	89438597	*ANKRD11*	5'UTR	0.18	n	0
cg08822215	ILMN_2108709	11.47	2.5e-02	8.75	3.0e-02	-14.03	2.4e-02	0.18	16	89438651	*ANKRD11*	5'UTR	-0.09	n	0
cg08822215	ILMN_1690465	-8.8	3.6e-04	-12.52	3.7e-04	14.88	3.8e-04	0.17	16	89438651	*ANKRD11*	5'UTR	-0.05	n	0
cg01881136	ILMN_1690465	-9.1	1.6e-03	-28.38	2.1e-03	30.59	2.1e-03	0.19	16	89438758	*ANKRD11*	5'UTR	0.1	n	0
cg09537792	ILMN_1690465	-8.49	8.0e-03	-21.08	8.8e-03	23.18	8.8e-03	0.11	16	89438892	*ANKRD11*	5'UTR	0.07	n	0

cg08417142	ILMN_1690465	-9.88	2.5e-04	-21.6	2.9e-04	25.4	2.9e-04	0.19	16	89439771	*ANKRD11*	5'UTR	0.2	n	0
cg08549241	ILMN_1690465	-8.03	2.3e-02	-8.35	2.7e-02	12.4	2.7e-02	0.1	16	89440133	*ANKRD11*	5'UTR	0.17	n	0
cg05638739	ILMN_1690465	-10.44	4.4e-05	-12.94	5.4e-05	18	5.3e-05	0.22	16	89440324	*ANKRD11*	5'UTR	0.19	y	0
cg16525838	ILMN_1690465	-8.56	4.9e-03	-11.02	5.7e-03	14.94	5.7e-03	0.12	16	89440460	*ANKRD11*	5'UTR	0.17	n	0
cg00770754	ILMN_1690465	-16.53	5.2e-03	-16.08	5.0e-03	22.83	5.1e-03	0.11	16	89440949	*ANKRD11*	5'UTR	-0.02	n	0
cg26946905	ILMN_1690465	-8.31	3.3e-03	-15.14	3.7e-03	17.7	3.7e-03	0.13	16	89441595	*ANKRD11*	5'UTR	0.07	n	0
cg02582213	ILMN_1690465	-7.22	8.0e-03	-13.71	1.0e-02	15.28	1.0e-02	0.16	16	89441654	*ANKRD11*	5'UTR	-0.01	n	0
cg00926926	ILMN_1690465	-10.57	2.9e-04	-28.55	3.6e-04	31.88	3.5e-04	0.19	16	89445433	*ANKRD11*	5'UTR	0.16	n	0
cg08595667	ILMN_1690465	-7.38	8.7e-04	-21.33	1.3e-03	23.24	1.3e-03	0.21	16	89445453	*ANKRD11*	5'UTR	0.11	n	0
cg00488747	ILMN_1690465	-9.12	1.2e-03	-17.53	1.5e-03	20.63	1.5e-03	0.16	16	89445503	*ANKRD11*	5'UTR	0.12	n	0
cg03484438	ILMN_1690465	-7.55	5.0e-03	-11.85	7.4e-03	14.59	7.3e-03	0.21	16	89452237	*ANKRD11*	5'UTR	0.12	n	0
cg02013146	ILMN_1690465	-10.27	1.3e-03	-26.97	1.4e-03	29.48	1.4e-03	0.15	16	89452259	*ANKRD11*	5'UTR	0.07	n	0
cg09823077	ILMN_1690465	-6.91	1.2e-02	-15.37	1.6e-02	17.62	1.6e-02	0.13	16	89452360	*ANKRD11*	5'UTR	0.14	n	0
cg06270206	ILMN_1690465	-6.71	1.7e-02	-11.32	2.3e-02	14.8	2.2e-02	0.13	16	89455108	*ANKRD11*	5'UTR	0.32	n	0
cg02226192	ILMN_1746706	-8.33	2.6e-02	-3.23	1.6e-02	8.2	2.8e-02	0.15	16	89461734	*ANKRD11*	5'UTR	-0.18	n	0
cg06365976	ILMN_1690465	-6.42	9.6e-03	-17.4	1.3e-02	17.94	1.4e-02	0.17	16	89462292	*ANKRD11*	5'UTR	-0.08	n	0
cg07118196	ILMN_1690465	-9.88	3.0e-03	-16.08	3.1e-03	20.29	3.2e-03	0.12	16	89462418	*ANKRD11*	5'UTR	0.18	n	0
cg05900234	ILMN_1690465	-10.5	2.4e-03	-17.94	2.7e-03	22.35	2.7e-03	0.13	16	89462661	*ANKRD11*	5'UTR	0.19	n	0
cg03258727	ILMN_1690465	-6.69	2.0e-02	-10.94	2.4e-02	12.88	2.4e-02	0.12	16	89462782	*ANKRD11*	5'UTR	0.04	n	0
cg07746351	ILMN_1690465	-11.51	1.9e-03	-17.99	2.1e-03	22.41	2.1e-03	0.15	16	89466392	*ANKRD11*	5'UTR	0.12	n	0
cg04340114	ILMN_1690465	-6.21	2.5e-02	-12.95	3.1e-02	14.76	3.1e-02	0.11	16	89468673	*ANKRD11*	5'UTR	0.09	n	0
cg00206490	ILMN_1746706	-9.67	3.8e-02	-1.98	1.9e-02	9.54	3.4e-02	0.17	16	89469729	*ANKRD11*	5'UTR	-0.27	n	0
cg09293925	ILMN_1690465	-5.84	1.5e-02	-10.92	1.6e-02	13.01	1.6e-02	0.08	16	89471650	*ANKRD11*	5'UTR	0.13	n	0
cg05749855	ILMN_1690465	-8.36	4.2e-03	-22.13	5.3e-03	24.35	5.3e-03	0.15	16	89472771	*ANKRD11*	5'UTR	0.1	n	0
cg09486407	ILMN_1690465	-10.93	4.4e-04	-20.13	5.7e-04	24.75	5.6e-04	0.2	16	89478268	*ANKRD11*	5'UTR	0.21	n	0
cg09222577	ILMN_2108709	10.15	3.7e-02	6.2	4.6e-02	-11.88	3.6e-02	0.17	16	89480222	*ANKRD11*	5'UTR	-0.04	n	0
cg09222577	ILMN_1690465	-6	3.9e-02	-6.87	3.9e-02	9.39	4.0e-02	0.06	16	89480222	*ANKRD11*	5'UTR	0.07	n	0
cg09222577	ILMN_1746706	7.38	3.6e-02	5.96	4.4e-02	-9.88	3.5e-02	0.13	16	89480222	*ANKRD11*	5'UTR	0.04	n	0

cg09971549	ILMN_1690465	-6.66	2.1e-02	-3.56	2.3e-02	7.68	2.3e-02	0.09	16	89487322	*ANKRD11*	5'UTR	0.08	n	0
cg26547698	ILMN_2108709	-11.9	4.4e-02	-1.96	1.7e-02	12.03	4.4e-02	0.16	16	89489058	*ANKRD11*	5'UTR	0	n	0
cg26547698	ILMN_1690465	-8.59	1.2e-02	-2.3	1.2e-02	9.24	1.2e-02	0.09	16	89489058	*ANKRD11*	5'UTR	0.15	n	0
cg26547698	ILMN_1746706	-10.06	2.2e-02	-2.08	9.3e-03	10.2	2.2e-02	0.14	16	89489058	*ANKRD11*	5'UTR	-0.02	n	0
cg06489433	ILMN_1690465	-7.75	1.1e-02	-15.5	1.5e-02	17.53	1.5e-02	0.2	16	89491229	*ANKRD11*	5'UTR	0.05	n	0
cg09640686	ILMN_1690465	-7.92	8.7e-03	-12.66	1.1e-02	15.26	1.1e-02	0.14	16	89496981	*ANKRD11*	5'UTR	0.07	n	0
cg09907628	ILMN_1690465	-8.66	3.8e-03	-9.11	5.1e-03	12.91	5.0e-03	0.19	16	89498634	*ANKRD11*	5'UTR	0.09	n	0
cg02192318	ILMN_1690465	-6.96	3.9e-02	-11.05	4.6e-02	13.19	4.6e-02	0.11	16	89500212	*ANKRD11*	5'UTR	0.05	n	0
cg12914530	ILMN_1690465	-9.91	1.5e-04	-21.03	1.7e-04	24.08	1.7e-04	0.19	16	89500225	*ANKRD11*	5'UTR	0.1	n	0
cg06039074	ILMN_1746706	-10.51	2.8e-02	-1.4	9.7e-03	10.6	2.4e-02	0.21	16	89502388	*ANKRD11*	5'UTR	-0.22	n	0
cg16765487	ILMN_1690465	-7.53	7.8e-03	-14.06	8.8e-03	16.41	8.9e-03	0.11	16	89548175	*ANKRD11*	5'UTR	0.08	n	0
cg05288253	ILMN_1690465	-6.82	1.8e-02	-4.43	1.8e-02	8.47	1.9e-02	0.08	16	89552259	*ANKRD11*	5'UTR	0.1	n	0
cg08438650	ILMN_2108709	-9.16	4.2e-02	-3	2.4e-02	9.36	4.3e-02	0.16	16	89558207	*ANKRD11*	TSS1500	-0.05	n	0
cg16958716	ILMN_1708508	-6.98	3.2e-02	-1.34	1.5e-02	7.07	3.1e-02	0.1	17	56833425	*PPM1E*	1stExon	-0.04	n	0
cg04662983	ILMN_1708508	-9.33	6.8e-03	-1.88	3.2e-03	9.43	5.8e-03	0.16	17	56834321	*PPM1E*	Body	-0.14	y	0
cg22088743	ILMN_2133675	9.56	4.7e-03	16.9	3.5e-03	-18.97	3.7e-03	0.19	17	78183317	*SGSH*	3'UTR	-0.06	y	0
cg09902913	ILMN_2133675	5.48	4.7e-02	18.8	3.6e-02	-19.85	3.7e-02	0.14	17	78184432	*SGSH*	Body	0.05	n	0
cg06331359	ILMN_2133675	6.35	1.9e-02	7.79	1.5e-02	-10.49	1.6e-02	0.11	17	78190755	*SGSH*	Body	0.09	n	0
cg03031613	ILMN_1855910	10.43	3.0e-02	2.93	2.4e-02	-10.61	2.8e-02	0.09	18	55018587	*ST8SIA3*	TSS1500	-0.12	n	0
cg03031613	ILMN_1697301	4.55	4.0e-02	1.57	3.0e-02	-4.91	3.5e-02	0.08	18	55018587	*ST8SIA3*	TSS1500	-0.01	n	0
cg13096208	ILMN_1855910	9.05	4.6e-03	1.49	3.1e-03	-8.63	6.4e-03	0.24	18	55019843	*ST8SIA3*	1stExon	-0.11	y	0
cg00866814	ILMN_1668194	-5.78	7.0e-03	-2.98	9.1e-03	6.46	8.4e-03	0.11	19	49017364	*LMTK3*	TSS1500	0.03	y	0
cg00789528	ILMN_2082209	-9.46	2.6e-02	-2.41	1.8e-02	9.71	2.8e-02	0.13	20	42543034	*TOX2*	TSS1500	0.06	n	0
cg26365090	ILMN_2082209	-10.07	1.2e-02	-0.55	2.7e-03	10.11	1.3e-02	0.13	20	42574362	*TOX2*	5'UTR	0.21	y	0
cg09628707	ILMN_1761903	7.53	8.7e-03	3.09	9.0e-03	-7.86	9.7e-03	0.11	20	43729410	*KCNS1*	5'UTR	-0.05	y	0

Beta: standardized regression coefficients

[a]: *correlation value between methylation and gene expression*

[b]: *knwon age-related CpG (No = 0; Yes =1)*

Supplementary Table S3 Literature-based assessment of the functional role of age-related eCpG genes in adipose tissue and obesity

Gene	Max. R2	Methylation	SNP	Transcript	Animal	Role (functional)	Notes	References
ANKRD11								
COL11A2	0.5	yes	yes	yes	human/pig	part of collagen involved in structure and strength of tissue support	1. epigenetically associated with MetS and 2. insulin resistence in visceral fat from obese subjects 3. upregulated in fat pig breed (JNP)	[36,37]
C1s	0.54			yes	human	part of complement system involved in inflammation	1. upregulated in adipose tissue in heavier BMI co-twins, 2. upreulated in adipose tissue in obese males	[38]
CPEB2								
TOX2								
KCNS1								
FHL2	0.31	yes	yes	yes	human/mouse	regulation of intracellular signal transduction pathways	1. hyper-methylation is associated with human aging and increase gene expression in blood and adipose, 2. FHL2 methylation in both blood and adipose associated with aging	[39]
TIAL1								
BSX								
PTPRN								

search terms "gene AND obesity", "gene AND adipose"
